# RePol: A high‐throughput screen for optimizing membrane protein solubilization and purification using polymers

**DOI:** 10.1002/pro.70407

**Published:** 2025-12-22

**Authors:** Adam Evans, Bethan Kelly, Pooja Sridhar, Alice J. Rothnie, Naomi L. Pollock, Philip M. Ireland, David I. Roper, Tim R. Dafforn

**Affiliations:** ^1^ School of Biosciences University of Birmingham Birmingham West Midlands UK; ^2^ Aston Institute of Membrane Excellence & School of Biosciences Aston University Birmingham UK; ^3^ Chemical, Biological and Radiological Division Defence Science and Technology Laboratory Wiltshire UK; ^4^ School of Life Sciences University of Warwick Coventry UK

**Keywords:** affinity resin, DIBMA, high‐throughput screen, immobilized metal‐ion affinity chromatography, membrane protein, optimization, protein purification optimization, SMA, synthetic polymer

## Abstract

Extraction and purification of membrane proteins has for a long time represented a significant challenge. Polymer‐based extraction methods, like those using styrene maleic acid co‐polymers have provided a fertile approach to generate samples that include the local lipid environment surrounding the protein. However, the wide variety of different polymers now available provides a challenge to identify the optimal solution. In this study we develop and demonstrate a novel high‐throughput screening approach for rapid optimization of polymer solubilization agents and chromatography resins for membrane protein purification. Using this approach, we explore whether there are standard conditions that perform well for a range of membrane protein morphologies, sources and functions. These data show that no such standard conditions exist for either polymer solubilization agent or chromatography resin and that some combinations are rarely suitable for membrane protein purifications under these conditions, such as the use of TALON resin at a pH of 7.5 or SMALP300 in the Synthetic Nanodisc Screening Kit MINI kit. Instead, the use of the screening approach developed in this work is the best route to an optimal membrane protein preparation protocol.

## 1 INTRODUCTION

Membrane proteins play a vital role in cellular function. Embedded in every lipid bilayer these amphipathic proteins are located with their hydrophobic regions integrated within or traversing across the bilayer to support cellular activity and viability. The presence of membrane proteins on the surface combined with their functions in controlling cellular homeostasis means these proteins are not only important for fundamental research but are also of central importance for drug and agrochemical discovery. Therefore, methods progressing the study of membrane proteins are of significant importance.

Conventionally, membrane proteins have been prepared for study by removing them from their native membrane by solubilizing them using detergents e.g. *n*‐dodecyl‐β‐D‐maltoside (DDM) (Ratkeviciute et al., 2021). These micelles mimic the lipid bilayer shielding the proteins' hydrophobic regions from the aqueous solvent (Seddon et al., 2004). Protein solubilization using detergents has a high rate of failure and when successful often generates samples with low activity and/or poor stability (Yang et al., 2014). Alternative approaches for membrane protein solubilization have been gaining popularity that aim to maintain native structure and function through preservation of the membrane surrounding the protein. Pioneers like Sligar et al., developed reagent systems based on peptides that allowed membrane proteins to be reinserted into nanodiscs which contained a lipid (Bayburt et al., 2002). However, these systems still relied on detergent during the initial protein extraction phase which fails to preserve the native membrane surrounding the protein (Ritchie et al., 2009). Building on this research, we developed the styrene maleic acid lipid particle (SMALP) in 2009 as the first method to preserve the native lipid environment intact around the protein after extraction (Knowles et al., 2009). The method was based on the use of an amphipathic polymer (styrene maleic acid co‐polymer) which excised 10 nm diameter discs of bilayer from the membrane including any resident protein. These now solubilized lipid‐membrane protein complexes are then able to be purified using standard chromatographic approaches. Since the development of this styrene maleic acid (SMA)‐based system there has been a rapid increase in the number of other polymer‐based reagents that can extract membrane proteins in a similar way (Akram et al., 2025; Hawkins et al., 2021; Rajan & Matsumura, 2015). Many of these have been developed to address known limitations of the original SMALP system such as low pH instability and susceptibility to divalent cations (Gulamhussein et al., 2019), but the relative performance of these polymers when compared to the original SMA is still in many cases, unclear. These developments have inevitably had positive effects on the membrane protein community, but they have also produced a “reagent landscape” which is complex for users and can be a barrier to the use of polymer‐based extraction technologies.

To resolve the issue of polymer reagent selection the research detailed here demonstrates the development of a rapid screening system to enable membrane protein scientists to select the most optimal polymer and affinity resin for their protein. The method is called the RePol screen for its ability to compare the compatibility between resin chromatography and polymer solubilization, which enables the choice of reagent systems for membrane protein extraction. The criteria used to develop the screening method are that it:

1. Can be carried out in a high throughput format (based on 96 wells).
2. Is quick and simple to use.
3. Has a low sample requirement reflecting the low availability of membrane protein targets.
4. Includes both screening of polymer and downstream chromatography types in recognition of the interactions between these two aspects of protein purification.
5. Has in‐built flexibility enabling different affinity chromatographic approaches to be swapped in and out of the protocol.
6. Has a detection method that quantifies both yield and purity of the sample after processing.

To address these criteria, the RePol system took inspiration from a method used in the screening of detergents (Raturi et al., 2023). This new method screens polymers that are commercially available and resins that are routinely used, enabling immediate use by the community but also providing a flexible system that can be extended as new polymers become available. It is anticipated that this approach will facilitate the optimization of purifying existing and novel synthetic polymer‐protein complexes. To demonstrate the utility of the RePol screen, screening data for a number of membrane proteins, including those of different functions and structural architectures and from different source membranes are presented. The generic applicability of the RePol screen is highlighted while at the same time providing the largest baselined dataset of polymer extraction performance against membrane protein types currently available. Although SMA has previously been shown to work well for proteins that express at relatively low levels (Bada Juarez et al., 2020; Patel et al., 2022), expression of the protein of interest should be optimized prior to high‐throughput methods that use minimal material. All the proteins of interest in this paper have previously had their expression levels optimized and most have previously been solubilized with SMA and SMA‐like co‐polymers (Dathe et al., 2019; Dörr et al., 2014; Pollock et al., 2019).

## 2 MATERIALS AND METHODS

### 2.1 Preparation of membranes containing membrane protein targets

#### 2.1.1 Bacterial targets expression

The genes for a selection of membrane proteins including *E. coli* ZipA, KcsA, *Y. pestis* PgsA, and *S. aureus* Sav1866 were expressed in various BL21 (DE3) derived *E. coli* strains. PgsA was expressed using BL21 (DE3) C41, ZipA was expressed using the BL21 (DE3) pLysS, KcsA and Sav1866 were expressed using the BL21 (DE3). Overnight cultures were made by picking and inoculating a single colony formed on LB agar supplemented with 100 μg/mL of Ampicillin from freshly transformed cells in 20 mL of LB media supplemented with 100 μg/mL of Ampicillin. The overnight culture was then diluted 1:100 into 2YT media for PgsA or LB media for ZipA, Sav1866 and KcsA. At an OD_600_ of 0.8, 0.4, 0.6 and 0.8 for PgsA, ZipA, Sav1866 and KcsA respectively, the cells were induced with 0.8 mM IPTG for PgsA or 0.5 mM IPTG for ZipA, Sav1866 and KcsA. PgsA, ZipA and Sav1866 were then expressed overnight at 18°C. KcsA was expressed at 37°C for 2 h. The cells were then harvested by centrifugation at 4000*g* for 20 min.

#### 2.1.2 Human embryonic kidney 296 (HEK293) targets expression

Lentiviral expression of Ntr1 was done using the HEK293 cell line. The HEK293 cells were cultured at 37°C and 8% [CO_2_] in Freestyle™ 293 Expression Medium (Gibco). Cells were passaged by a 1:10 dilution into fresh medium upon reaching a 2 × 10^6^ cells/mL density. The cells were harvested by centrifugation at 200*g* for 10 min.

#### 2.1.3 Membrane fraction isolation

The membrane fraction for *E. coli* cells expressing PgsA, ZipA, Sav1866 and KcsA was isolated by resuspending the cell membrane in lysis buffer (50 mM HEPES pH 7.5, 250 mM NaCl, 5% glycerol, 500 units Benzonase® Nuclease, 1 tablet cOmplete™, Mini, EDTA‐free Protease Inhibitor Cocktail) and lysing the cells with an Avestin Emulsiflex C3 homogenizer. The cells were passed through the homogenizer thrice while under 15,000–20,000 psi of pressure. The lysed cells were then centrifuged at 10,000*g* for 1 h. The supernatant was then aspirated from the pellet and transferred to an ultra‐centrifuge tube and centrifuged at 100,000*g* for 1 h to pellet the membrane fraction. The supernatant was then removed from the pelleted membrane fraction.

*E. coli* cells expressing Ntsr1 and HEK293 Ntr1 were lysed using an Avestin Emulsiflex C3 homogenizer at 20,000 or 10,000 psi respectively. The cell lysate was then centrifuged at 12,000*g* for 30 min. The supernatant was then aspirated from the pellet and transferred to an ultra‐centrifuge tube and centrifuged at 100,000*g* for 45 min.

At least 1000 mg wet weight of cell membrane was isolated for each target.

### 2.2 High throughput screening of polymer solubilization and affinity purification

#### 2.2.1 Solubilization screen

**FIGURE 1:**
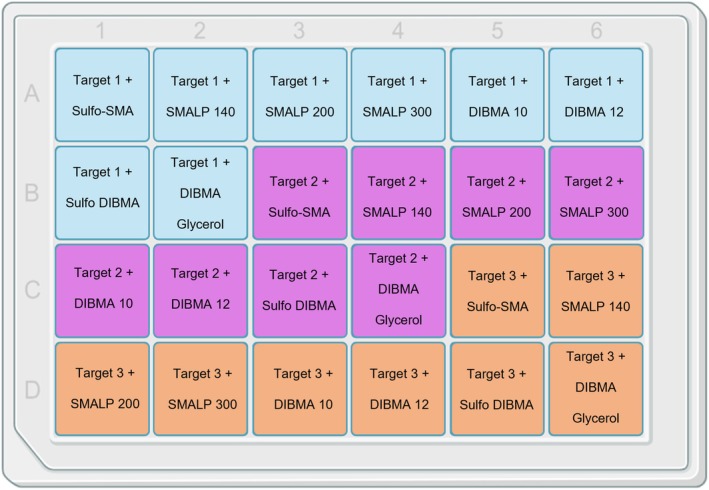
Up to three targets can be solubilized in each of the eight polymers from the Cube Biotech Synthetic Nanodisc Screening Kit MINI using a 24‐well Corning Deep well plate.

The membrane pellets containing the target protein were resuspended to give a final concentration of 100 mg/mL wet weight of cell membrane using membrane suspension buffer (50 mM HEPES pH 7.5 (NaOH), 500 mM NaCl, 5% glycerol). The polymers in the Cube Biotech Synthetic Nanodisc Screening Kit MINI were each dissolved in 1 mL of a 500 mM NaCl solution to produce the solubilization buffer (50 mM HEPES pH 7.5 (NaOH), 500 mM NaCl, 5% (w/v) polymer). Into a 24 well deep well plate (Corning Deep well plate, 24 well AXYPDW10ML24C) (see Figure 1), 1 mL of the resuspended membrane pellet was added into 8 of the wells. This was repeated twice more for a total of 3 different protein targets per 24‐well block. One milliliter of solubilization buffer was then added to each well (one polymer from the screen per well). The solubilization buffer (containing the relevant polymer) was then mixed with the membrane sample already in the well by gentle agitation at 450 rpm on a Starlab Microplate Mixer. Therefore, each well held a 2 mL final volume containing 2.5% (w/v) polymer and 50 mg/mL membrane concentration. The samples were left to solubilize for 2 h on an orbital shaker at room temperature. The samples were centrifuged in the plate at 2250*g* for 20 min to precipitate any aggregates.

#### 2.2.2 Chromatography resin screen

**FIGURE 2:**
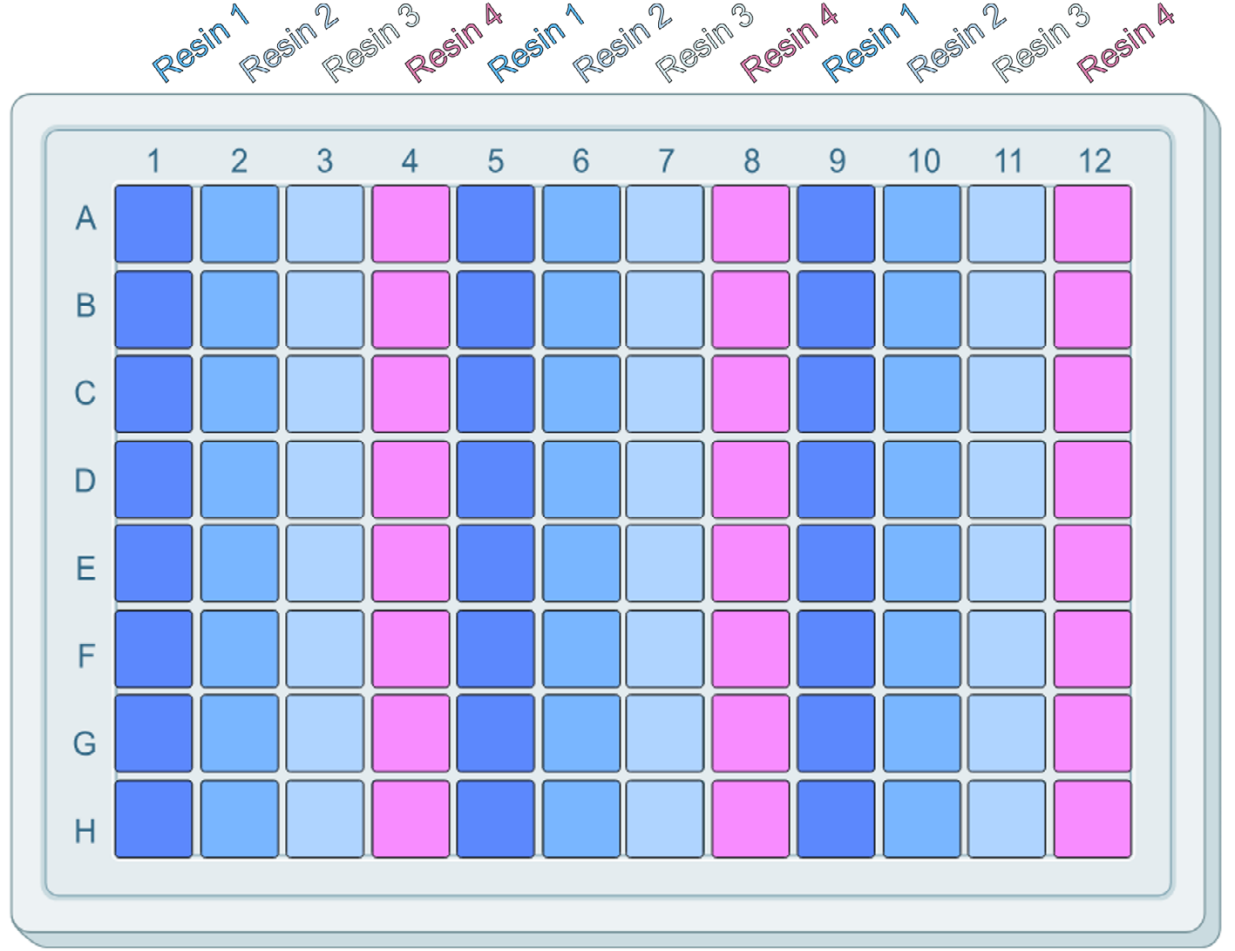
96‐Well block layout for resin. This layout is designed for up to three targets being solubilized in eight different conditions and screened against four resin types.

**FIGURE 3:**
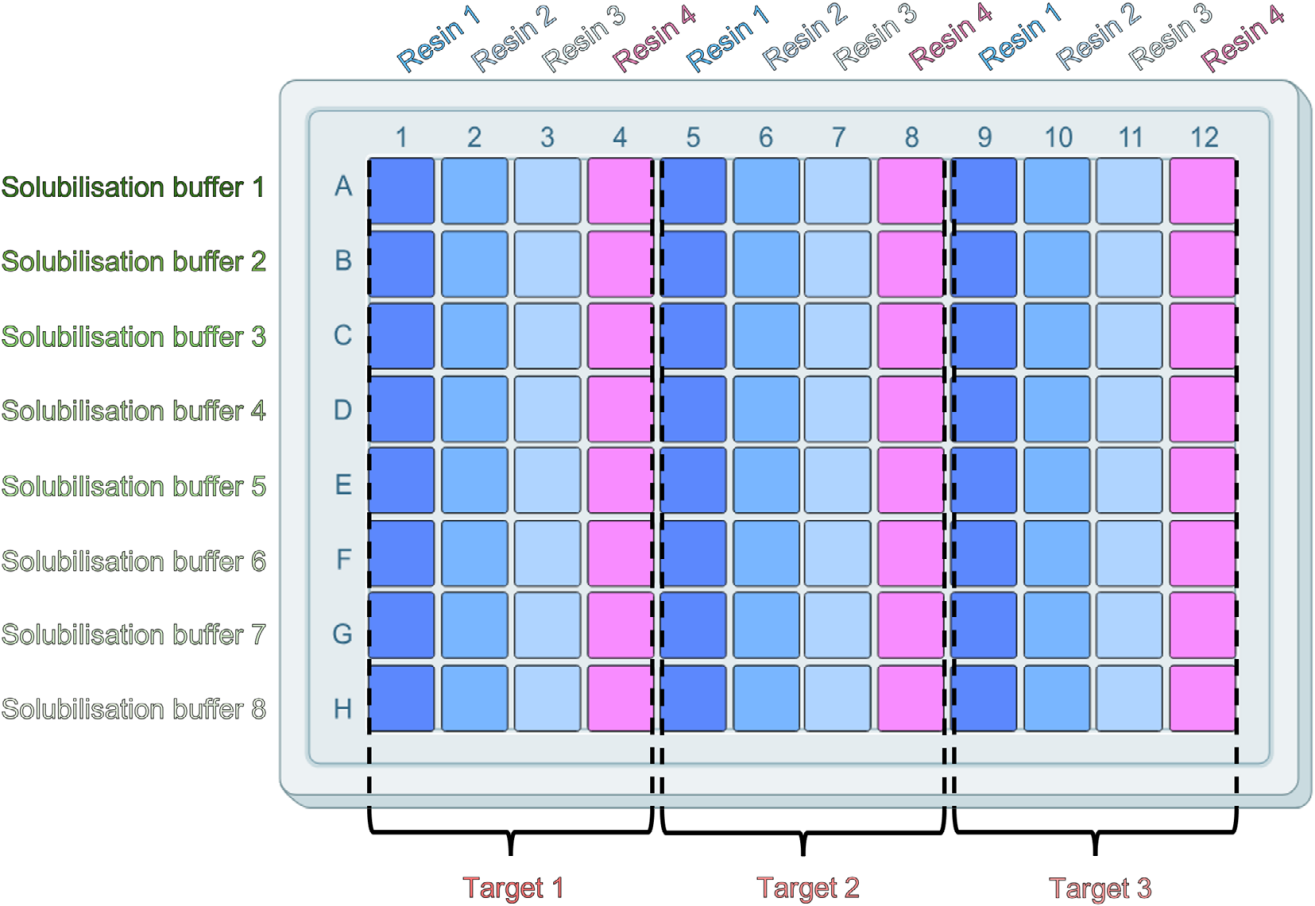
Resin—sample binding layout showing the division of the solubilized targets across each of the resin types.

The resins were pre‐equilibrated in membrane suspension buffer (50 mM HEPES pH 7.5 (NaOH), 500 mM NaCl, 5% glycerol) following the manufacturer's guidelines to ensure none of the storage buffer was present during the screening process. A 96 well deep well plate (VWR, Deep well‐plates square well, V‐bottom) was prepared that contained 100 μL bed volume of the resins screened (Figures 2 and 3). An affinity resin was used for binding to polyhistidine‐tagged proteins, but similar protocols can be developed for other affinity systems (e.g., FLAG, Strep‐Tactin, Biotin). Five hundred microliters of the solubilized membrane (taken from the relevant well in the solubilization screen) were transferred to the corresponding wells in the chromatography screen. If precipitate formed during the 2‐h solubilization incubation, an additional volume of membrane suspension buffer can be added to each well to ensure an even amount of sample can be distributed between the four resin types. If this additional volume is added, the same volume must be added to each solubilization condition, prior to being split across the resin types, regardless of the quantity of precipitate formed to ensure a comparable study. The plate was sealed using Molecular Dimensions EasySeal™ Sheets and left to incubate overnight at 4°C with constant agitation on a Starlab Microplate Mixer to ensure the resin remained suspended in each solution. It should be noted that a high RPM on an orbital shaker is required to ensure the resin is fully suspended in solution due to the narrow well size. The plate was then centrifuged at 600*g* for 15 min to sediment the resin. The supernatant was aspirated from the top, ensuring the resin pellet was not disturbed. This material can be reserved for later analysis as the unbound fraction. The resin was then washed by applying 1 mL of wash buffer (50 mM HEPES, pH 7.5 (NaOH), 500 mM NaCl, 5% glycerol, 20 mM imidazole) to each well, followed by gently pipetting up and down to fully resuspend the resin. The plate was then centrifuged again at 600*g* for 5 min to pellet the resin, followed by removal of the supernatant. Wash, sedimentation and supernatant removal steps were repeated two further times until the resin was washed with a total of 4 mL or 40 bed volumes of wash buffer. On the last wash step, 900 μL of the wash buffer was removed and the resin resuspended in the remaining 100 μL. The resuspended resin was then transferred to a new 96‐well filter plate (Pall Life Sciences 96 Well Filter Plate Acroprep Advance 2 mL 0.2 μm PTFE membrane) maintaining the indexing of the sample on the plate plan. The plate was washed with 100 μL additional wash buffer per well to collect the resin. The filter plate containing the resin was placed on top of a 96 well deep well plate and centrifuged at 600*g* for 5 min to ensure no wash buffer was remaining. The filter plate containing the resin was then placed onto a clean 96 well microtiter plate. One hundred microliters of elution buffer (50 mM HEPES, pH 7.5 (NaOH), 500 mM NaCl, 5% glycerol, 250 mM imidazole) was pipetted into each well and incubated on a Starlab Microplate Mixer for 10 min at room temperature at approximately 450 rpm. The stacked plates were then centrifuged at 600*g* for 5 min. The sample centrifuged into the clean 96 well plate contains the protein eluted from the resin and was labeled Eluted Samples. The eluted samples were then either frozen for future use at −20°C or run on SDS‐PAGE for analysis.

#### 2.2.3 Comparison with n‐dodecyl‐β‐D‐maltoside membrane protein solubilization

The procedure for the n‐dodecyl‐β‐D‐maltoside (DDM) baseline was performed as described above, but with a few minor changes. The solubilization buffer contained a 2% DDM solution instead of the 5% polymer. Each subsequent buffer contained 0.51 mM DDM to ensure the concentration of DDM did not drop below the critical micelle concentration (Table 1).

**TABLE 1:** Summary of the synthetic nanodisc screening kit MINI, purchased from Cube Biotech detailing the physical characteristics of each polymer.

	SMALP 140	SMALP 200	SMALP 300	Sulfo‐SMA	DIBMA 10	DIBMA 12	DIBMA glycerol	DIBMA glucosamine	DDM
Molecular weight	5 kDa	6.5 kDa	10 kDa	9 kDa	10 kDa	12 kDa	10 kDa	10/12 kDa	0.51 kDa
Divalent cation tolerance	<5 mM	<5 mM	<5 mM	>100 mM	<5 mM	<5 mM	<50 mM	<50 mM	>50 mM
Solubility in H_2_O	45%	40%	35%	>10%	>10%	>10%	>10%	>10%	>10%
CMC in H_2_O	N/A	N/A	N/A	N/A	N/A	N/A	N/A	N/A	0.17 mM

### 2.3 Polyacrylamide gel electrophoretic analysis of samples

Fifteen microliters of each sample from the Eluted Samples plate was added to 5 μL of reducing SDS‐PAGE loading dye (NuPAGE™ LDS Sample Buffer (4X) + 10 mM DTT). Ten microliters of this was then loaded into an SDS‐PAGE gel (4%–15% Criterion™ TGX™ Precast Midi Protein Gel, 26 wells), with each protein target flanked by a protein ladder marker and run until resolved. The samples were not boiled to avoid aggregation. The gels were run in SDS running buffer (2.5 mM Tris pH 8.3, 19.2 mM glycine, 0.01% w/v SDS) until the pre‐stained ladder was well resolved and were then stained using Coomassie quick stain for 1 h to overnight, destained in distilled water to remove background dye, and imaged using the GE Amersham Imager AI680.

### 2.4 Western blot analysis of samples

Immunoblotting was carried out as described per Pollock et al. (2019) with minor changes. The polyacrylamide gels were run as described above and were transferred onto a 0.2 μm PVDF membrane using the Trans‐Blot Turbo Transfer System and Trans‐Blot Turbo Midi 0.2 μm PVDF Transfer Packs from Bio‐Rad. The transfer was performed as described in the Trans‐Blot Turbo Transfer System Instruction Manual.

### 2.5 Data analysis

The target of interest will migrate to a region on the gel in accordance with its molecular weight. However, it is important to note that slight variations may occur due to gel shift (Rath et al., 2009). The gel was visually inspected to determine the lane in which the band at the expected position is most dense. Band densitometry data were attained for KcsA as an example, and was done using ImageJ 1.54 g software as described by Stael et al. (2022).

## 3 RESULTS

### 3.1 Choice of polymer

There are currently more than 20 polymers related to SMA and DIBMA commercially available to researchers. While it is possible to apply this screening technology to test all these polymers, we chose to use a smaller number of polymers in this study to allow a wider range of proteins to be tested enabling the performance of polymers across a range of protein classes to be assessed. We therefore chose to use a pre‐existing screen from Cube Biotech called the “Synthetic Nanodisc Screening Kit MINI” which contains eight different polymers (Table 1), of which four are variations of SMA and four are variations of DIBMA. This provides an assessment of the most commonly used variants as well as a number of newer polymers that are thought to offer advantages in solubilization. These newer polymers often attempt to mitigate some of the more limiting factors of using SMA and SMA‐like copolymers, such as the size limitations of the protein complex able to be solubilized, low divalent cation tolerance and low pH sensitivity (Lee et al., 2016). Alongside samples containing polymers a control sample based on existing methods (Raturi et al., 2023) was included which used the detergent DDM and TALON resin to allow samples to be baselined against a common detergent purification method.

### 3.2 Choice of resin

It is well established that the type of His‐affinity resin used to purify a protein can influence both the yield and purity (Valeria et al., 2020). This is also observed for polymer solubilizations, so different resins were included in the screen to allow researchers to assess the influence of resin type and polymer in combination. Resins were chosen that included different chelating groups (nitrilotriacetic acid, iminodiacetic acid, and carboxymethylated aspartate) alongside different support types (cellulose and silica). The details of each resin are shown in Table 2.

**TABLE 2:** Summary of some of the physicochemical characteristics of the resins used for this screen.

	Ni‐NTA Superflow	Ni‐IDA	Ni‐sepharose high performance	TALON
Metal ion	Nickel	Nickel	Nickel	Cobalt
Chelating agent	Nitrilotriacetic acid (NTA)	Iminodiacetic acid (IDA)	Nitrilotriacetic acid (NTA)	Carboxymethylated aspartate
Coordination number for metal	4	3	4	4
Resin matrix	Superflow (sepharose)	Silica	Sepharose	Sepharose
Maximum binding capacity	50 mg/mL	10 mg/mL	40 mg/mL	15 mg/mL
Bead size	60–160 μm	Unknown	34 μm	45–165 μm
Company	Qiagen	Macherey‐Nagel Protino	Cytiva	Takara

### 3.3 Further tunable parameters

If no ideal conditions are found, other variables can be screened, such as the buffer composition, wash and elution conditions and choice and concentration of solubilization agent (detergent, AASTY's or Ultrasolute™ Amphipols). This method allows much wider variations than those showcased in this study, but the ease and simplicity the Cube Synthetic Nanodisc Screening Kit MINI provides was ideal for highlighting the necessity of screening synthetic polymers against various resin types. Following the protocol of the Cube Synthetic Nanodisc Screening Kit MINI produced a final synthetic polymer concentration of 2.5% for all synthetic polymers used in this screen. Previous publications show that to achieve similar levels of solubilization as SMALP 200, higher concentrations of DIBMA‐based synthetic copolymers may be required (Grethen et al., 2017; Gulamhussein et al., 2020).

### 3.4 Optimization of prior steps

Many conditions of the purification process can be optimized by retaining various stages of this high‐throughput purification method. For example, if a sample of the membrane fraction is taken prior to solubilization, it could be used to compare the solubilization efficiency of each synthetic polymer. For another example, if the flowthrough of the wash buffer is retained, the concentration of imidazole, for IMAC purifications, could be adjusted to optimize the ratio of contaminants and target protein eluted during this wash step. However, this falls outside the purview of this paper, as we aim to highlight the significance of the RePol screen, and its core concept of identifying the best combination of resin and polymer, and how this is a protein‐dependent characteristic.

### 3.5 Selection of protein target

Screening was carried out using seven integral membrane proteins from several expression hosts. This ensured that the screening protocol was generically applicable across protein target types and membrane sources. In addition, it allowed a wider analysis of resin and polymer side‐by‐side performance. The repertoire of proteins used in the study was determined by the availability of relevant proteins that carried a polyhistidine tag and was curated to generate a diverse set. The aim was to understand whether common protein architectures behaved similarly and whether similar proteins expressed in different membrane systems behaved similarly. The protein set also included representatives of families with significant interest to academic and commercial biochemists including two G‐protein‐coupled receptors, an ABC transporter, and an ion channel (Table 3).

**TABLE 3:** Protein targets used during the study, highlighting the expression system, source organism and number of transmembrane domains of each target.

Protein target	Protein name and/or class	Expression system	Source organism	Number of TM domains	Tags
PgsA	Transferase	*Escherichia coli*	*Yersinia pestis*	12 (Yang et al., 2021)	9×His, SUMO, FLAG, 3C
ZipA	Membrane Tether	*Escherichia coli*	*Escherichia coli*	1 (Mosyak et al., 2000)	6×His
SAV1866	ABC Transporter	*Escherichia coli*	*Staphylococcus aureus*	12 (Dawson & Locher, 2007)	6×His
KcsA	Potassium Ion Channel	*Escherichia coli*	*Streptomyces lividans*	8 (MacKinnon, 2003)	6×His
Ntsr1	Neurotensin Receptor/GPCR	*Escherichia coli*	*Rattus norvegicus*	7 (White et al., 2012)	10×His, ThioRed
Ntr1	Neurotensin Receptor/GPCR	Human embryonic kidney	*Homo sapiens*	7 (Egloff et al., 2014)	10×His, FLAG

#### 3.5.1 RePol screen for assessing polymer‐resin performance for ZipA extraction and purification

**FIGURE 4:**
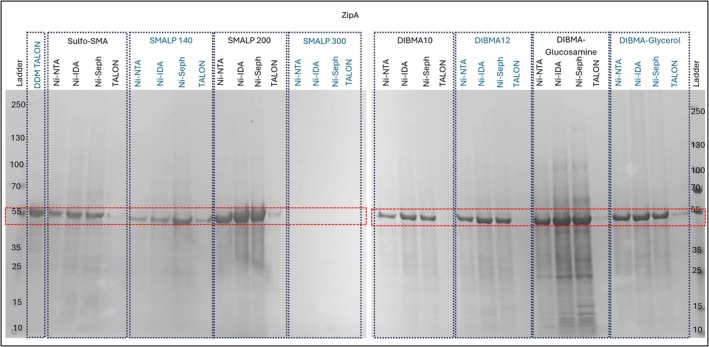
RePol screen results of ZipA resolved on a 4%–15% gradient Tris/glycine SDS‐PAGE gel stained with Coomassie quick stain. DDM TALON, DDM solubilized target, extracted using TALON from Takara; Ni‐IDA, Ni‐IDA from Macherey‐Nagel Protino; Ni‐NTA, Ni‐NTA Superflow from Qiagen; Ni‐Seph, Ni‐sepharose high performance from Cytiva; TALON, TALON from Takara.

ZipA is a topologically simple membrane protein which was one of the first to be extracted by an SMA‐based polymer extraction system (Teo et al., 2019). ZipA is a membrane protein from the *E. coli* inner membrane which is involved in cell division. It contains an N‐terminal transmembrane domain which consists of a single transmembrane α‐helix. This is connected to a soluble folded domain at the C‐terminal by an intrinsically unstructured domain (Lee et al., 2019). The protein used in this study is expressed in *E. coli* with a C‐terminal 6‐His tag. The protein is known to be amenable to extraction by a range of polymers (Morrison et al., 2016) and its high yield makes it ideal for initial testing and optimization of the RePol Screen. The format of the screen means that a single protein like ZipA requires 32 lanes of a PAGE gel for each polymer/resin combination, plus additional lanes for a ladder marker and DDM‐TALON control (Figure 4). For ease of analysis the position the target protein would be expected to migrate to is indicated by a dashed box on the SDS‐PAGE figures. The DDM solubilized fraction (extracted by TALON resin) is shown as the first lane after the markers. The rest of the lanes are organized into blocks of four samples which represent the eluted fractions from each of the four test resins for one polymer type. It should be noted that for brevity only the soluble fraction after extraction and elution is shown. If required, it is possible to analyze samples using PAGE at other stages in the screening process to probe solubilization separately. For these samples, which generally contain a wide range of proteins alongside the target, a specific detection technique like Western blot is required for assessment of target levels.

Analysis of the results from the RePol screen for ZipA (Figure 4) shows that for a wide range of conditions, measurable amounts of ZipA could be extracted and separated to provide samples of differing levels of purity. The only polymer that failed to extract any measurable amounts of ZipA was SMALP 300 which contains a 3:1 ratio of styrene to maleic acid. This was unexpected given the successful use of similar 3:1 polymers in other studies (Morrison et al., 2016; Unger et al., 2021) and it will become clear later in the study that this polymer in this commercial screen behaves anomalously. A similar analysis of resin types shows that for three out of four of the resins, significant amounts of ZipA were bound and eluted generating samples of good purity. However, samples processed using the TALON resin generated very low levels of ZipA and in some cases (e.g., DIBMA10) no protein was apparent in the TALON sample. In contrast, the other three resins generated good protein yields with the same polymer. This suggests that TALON resins should not be used to purify ZipA when polymer extractions are used with these buffer conditions. Why TALON is unsuccessful is unclear but could be an incompatibility with the polymers used, the tag on the protein or the buffer conditions. This is analyzed further in Section 4. A more detailed analysis of these data shows that different polymer/resin combinations can influence yield in a more subtle way. For example, the SMALP 140 polymer does produce protein, but the yields are low compared to other polymers. While SMALP 200 and DIBMA‐glucosamine produce very good yields of the target, the DIBMA‐glucosamine samples contained significant levels of impurities which would require a second purification step that may not be necessary using SMALP 200. The use of SMALP 200 with either Ni‐sepharose high performance (hereafter referred to as Ni‐NTA sepharose HP) or Ni‐IDA provides the optimal combination for purity and yield for the preparation of ZipA. The relatively large number of conditions that yield pure ZipA might suggest that screening is not required. However, this dataset contrasts with the results attained from the RePol screening of the membrane protein PgsA.

**FIGURE 5:**
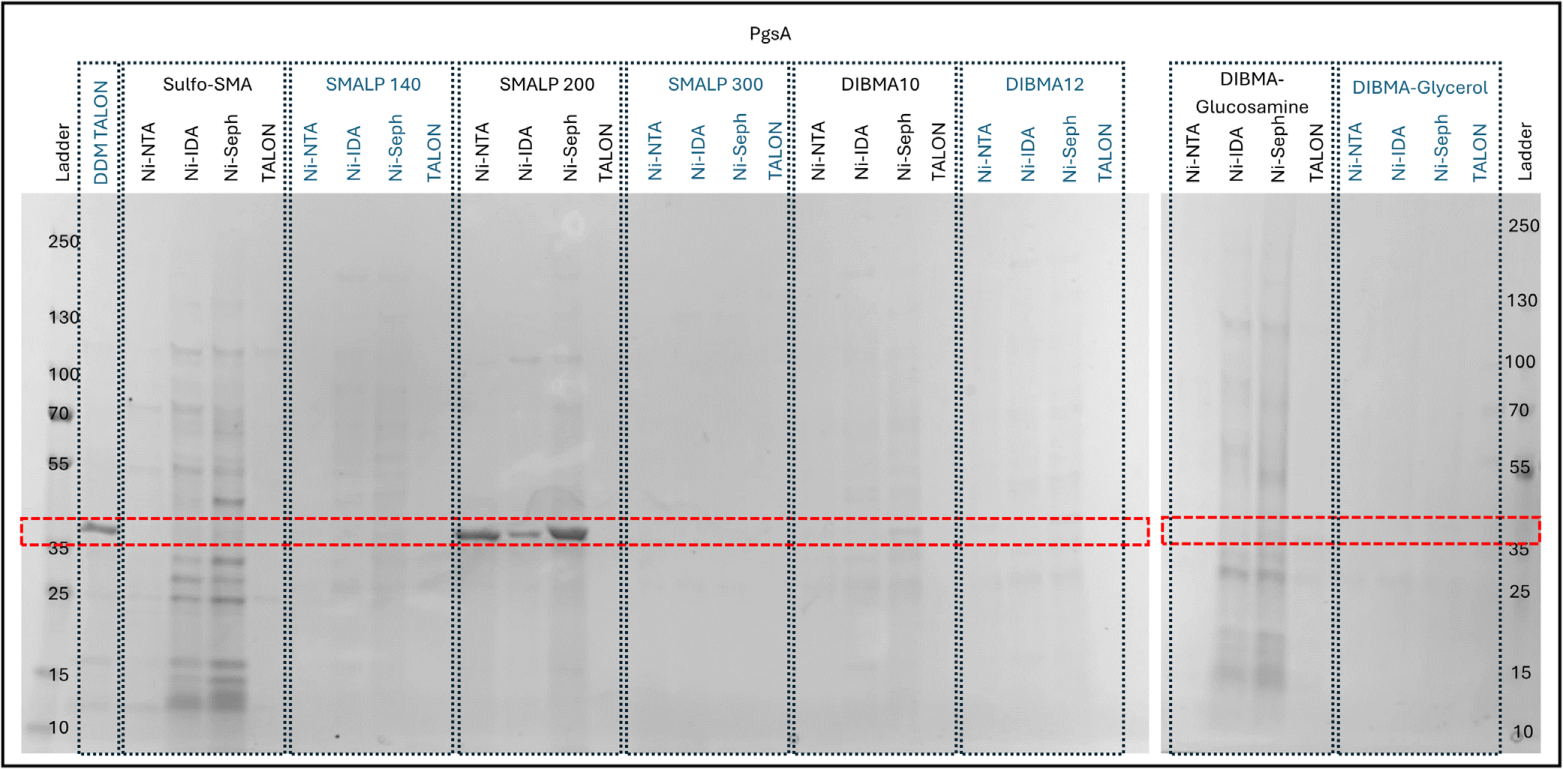
RePol screen results of PgsA resolved on a 4%–15% gradient tris/glycine SDS‐PAGE gel stained with Coomassie quick stain. DDM TALON, DDM solubilized target, extracted using TALON from Takara; Ni‐IDA, Ni‐IDA from Macherey‐Nagel Protino; Ni‐NTA, Ni‐NTA Superflow from Qiagen; Ni‐Seph, Ni‐sepharose high performance from Cytiva; TALON, TALON from Takara.

PgsA is an essential inner membrane protein that is responsible for the synthesis of phosphatidylglycerol phosphate (PGP) (Li et al., 2016; Yang et al., 2021). PgsA strongly favors a single polymer, SMALP 200, with DDM/TALON being the only other condition producing a distinct band within the expected region on the gel (Figure 5). Notably, the SMALP 200 solubilized condition purified with TALON produced no discernable bands. These data clearly demonstrate the utility of the RePol screen to optimize membrane protein extraction and purification using SMA and DIBMA‐like polymers.

#### 3.5.2 RePol screen for assessing polymer‐resin performance for Sav1866 extraction and purification

**FIGURE 6:**
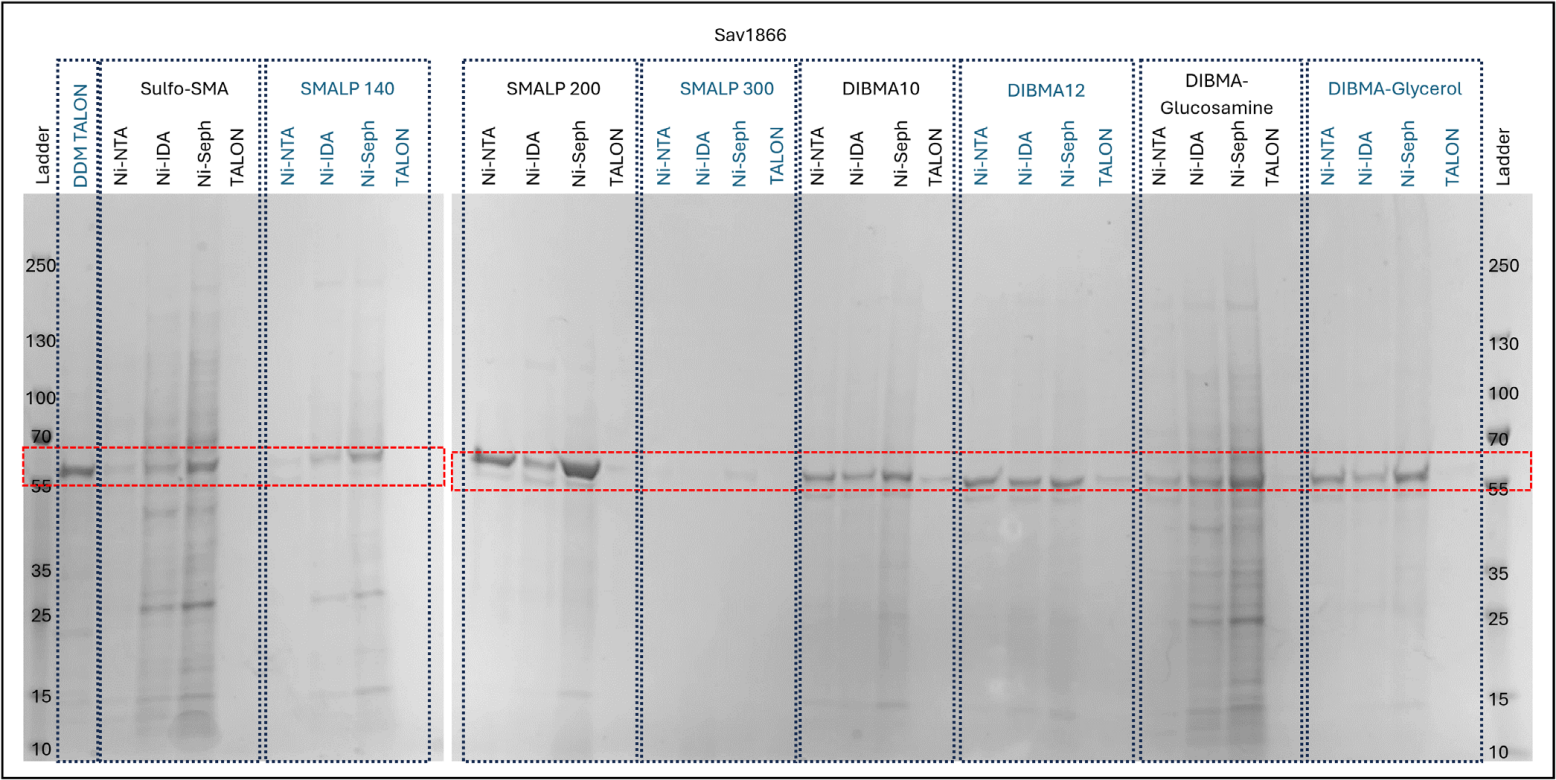
RePol screen results of Sav1866 resolved on a 4%–15% gradient tris/glycine SDS‐PAGE gel stained with Coomassie quick stain. DDM TALON, DDM solubilized target, extracted using TALON from Takara; Ni‐IDA, Ni‐IDA from Macherey‐Nagel Protino; Ni‐NTA, Ni‐NTA Superflow from Qiagen; Ni‐Seph, Ni‐sepharose high performance from Cytiva; TALON, TALON from Takara.

ZipA is in many ways not an archetypical membrane protein. It contains just a single transmembrane helix, is generally monomeric and the rest of the protein is extended away from the membrane by a long unstructured domain (Lee et al., 2019). In contrast, the bacterial ABC‐Transporter, Sav1866 has an extensive transmembrane domain (containing 12 transmembrane helices) is dimeric and has an extracellular domain that is very closely coupled to the transmembrane domain which is inherent to its molecular mechanism (Dawson & Locher, 2006, 2007). This makes Sav1866 divergent in structure from ZipA and allows the use of the RePol screen to assess if structural architecture has an influence on extraction and purification. As with ZipA, conditions containing SMALP 300 and/or TALON generated Sav1866 samples with low or no yield of the target (Figure 6). Comparison of those conditions which provided high yields of Sav1866 were similar but varied sufficiently to justify the use of a screening approach like RePol. Like ZipA, the combination of SMALP 200 and Ni‐NTA sepharose HP provided the highest yield with a good level of purity. However, unlike ZipA SMALP 200/Ni‐IDA generated smaller yields than SMALP 200/Ni‐NTA sepharose Superflow (hereafter referred to as Ni‐NTA sepharose SF). In addition, the yield from all the DIBMA‐glucosamine samples was greatly reduced compared to the results for ZipA. These results strongly suggest that protein architecture does influence the performance of different purification conditions. However, at least for ZipA and Sav1866, the SMALP 200/ Ni‐sepharose HP provides the best combination for both proteins.

#### 3.5.3 Does SMALP 200 and Ni‐NTA sepharose SF provide a universal solution for membrane protein purification?

**FIGURE 7:**
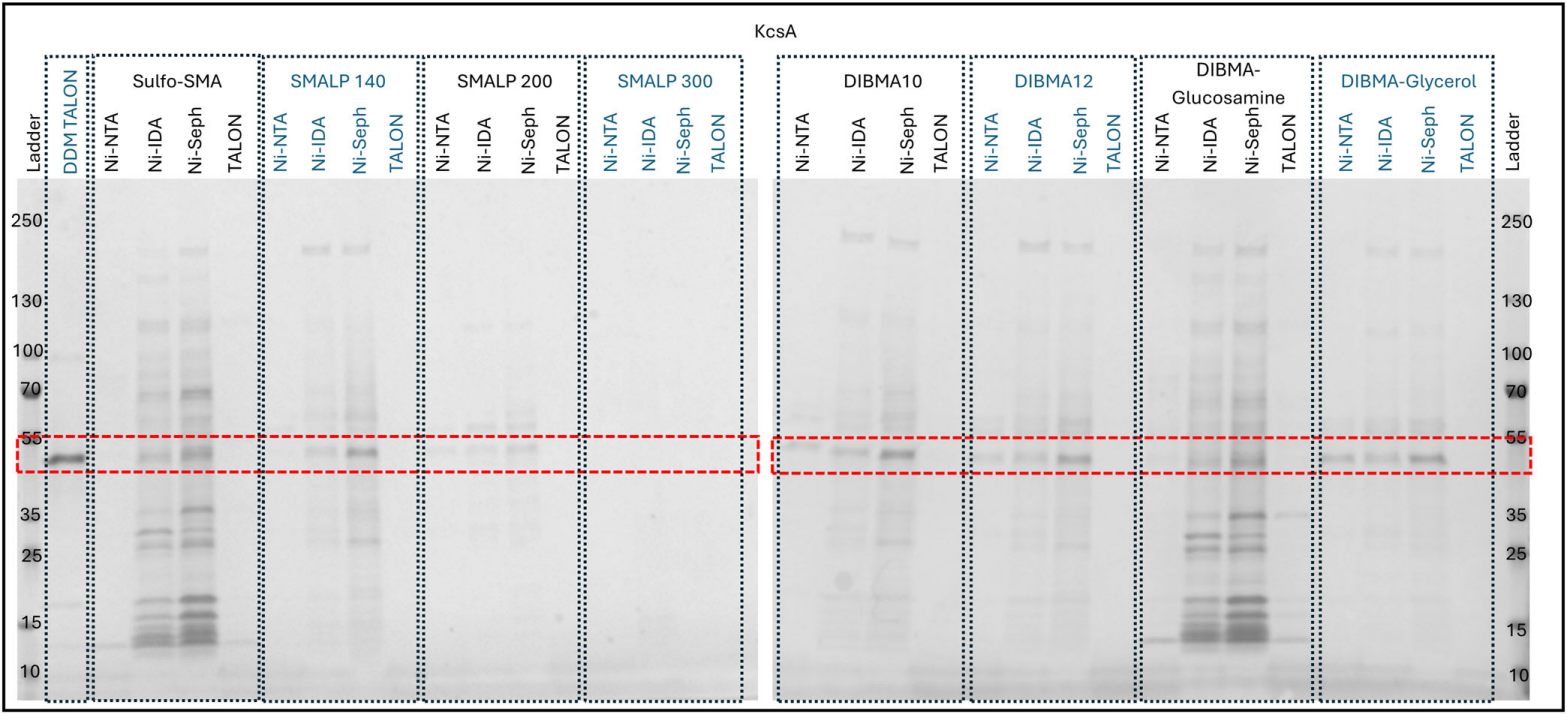
RePol screen results of KcsA resolved on a 4%–15% gradient tris/glycine SDS‐PAGE gel stained with Coomassie quick stain. DDM TALON, DDM solubilized target, extracted using TALON from Takara; Ni‐IDA, Ni‐IDA from Macherey‐Nagel Protino; Ni‐NTA, Ni‐NTA Superflow from Qiagen; Ni‐Seph, Ni‐sepharose high performance from Cytiva; TALON, TALON from Takara.

RePol data for ZipA and Sav1866 suggests that SMALP 200/Ni‐NTA sepharose SF provides a single condition that generates the highest yields for each protein. Given the structural diversity of these proteins, it could be concluded that this is true for all proteins. To test this hypothesis the RePol screen was applied to a third protein that is structurally and functionally divergent from both ZipA and Sav1866. KcsA is a tetrameric potassium channel with a total of eight transmembrane helices and short soluble linkers between its transmembrane domains. KcsA has higher hydrophobicity than the other two proteins tested, which each have soluble domains of significant size. Data from the RePol screen on KcsA clearly demonstrate that the SMALP 200/Ni‐NTA sepharose SF combination also generates some KcsA albeit with impurities present (Figure 7). To optimize the purity of a purification, once a condition that produces the most abundant protein of interest is found, wash steps and post IMAC processing can be optimized to refine the purity of the target. However, some methods such as size exclusion chromatography may not be feasible for all protein targets due to the nature of SMALPs normalizing the size of molecules in the solution to a 10 nm diameter disc. Examination of these data shows that at least four other conditions (sulfo‐SMA/Ni‐NTA sepharose HP, SMALP 140/Ni‐NTA sepharose HP, DIBMA10/Ni‐NTA sepharose HP, DIBMA12/Ni‐NTA sepharose HP, DIBMA‐glycerol/Ni‐NTA sepharose HP) provide greater yields of the target protein than the SMALP 200/Ni‐NTA sepharose HP combination. Interestingly, in each case the resin used, Ni‐sepharose HP, is common to that used for ZipA and Sav1866 but in each case the polymer is different. It is also worth noting that the polymers come from two different polymer classes, SMA and DIBMA. KcsA has previously been produced using polymer extraction methods (Dörr et al., 2014) using SMA2000 from Cray Valley/HisPur™ Ni‐NTA Superflow Agarose from ThermoFisher. Our data suggest that these conditions would have been generating suboptimal yields and use of the RePol screen could have provided conditions that increase yields significantly, as can be seen in Figure S1.

#### 3.5.4 Band densitometry of SDS‐PAGE gels

Band densitometry for KcsA was done using ImageJ 1.54g software as described by Stael et al. (2022). The KcsA bands from the KcsA SDS‐PAGE gels were identified, and the discernible peaks were labeled. As the total KcsA sample was split into the 32 samples, the percentage of KcsA purified per condition was calculated and plotted (see Figure S1a) to compare which condition purified the greatest quantity of KcsA, regardless of non‐target bands. This method shows that DIBMA‐glucosamine/Ni‐sepharose would produce the most KcsA. However, when comparing the six conditions previously mentioned that purified the most KcsA (sulfo‐SMA, SMALP140, DIBMA10, DIBMA12, DIBMA‐glucosamine and DIBMA‐glycerol using Ni‐sepharose resin), it is clear that the DIBMA‐glucosamine/Ni‐sepharose condition is the worst in regard to the KcsA to non‐specific protein ratio as 16 bands were identified, with KcsA being only 11.3% of the total within that condition. DIBMA10/Ni‐sepharose produced the largest proportion of KcsA to non‐specific protein (12 identifiable bands, with 48.5% being KcsA), and DIBMA‐glycerol/Ni‐sepharose gave the fewest non‐specific bands (7 identifiable bands, 45.5% being KcsA) (see Figure S1b). By combining the RePol methodology with band densitometry, the user of this method is able to specify if a large quantity of the target protein is desired over purity, or vice versa, depending on the application of the protein of interest.

#### 3.5.5 Do analogous proteins expressed in different cell lines share the same optimal resin and polymer combination?

To address this question, we looked at two GPCR homologs, Ntr1 and Ntsr1. Ntr1 is a human‐derived GPCR that was recombinantly expressed in the HEK293 cell line. Like all GPCRs it contains seven transmembrane α‐helices, and is thought to exist in vivo in a monomeric state, although some data suggests the presence of homo‐ and hetero‐oligomeric species (Jae Ryoung et al., 2010). To compare the expression systems, the homolog from *R. norvegicus* was expressed in *E. coli* cells. Also containing the standard seven transmembrane domains, the UniProtKB BLAST tool shows that there is an 84.21% sequence identity between the two homologs, with 93% of the transmembrane domains being identical. Additional differences in the mature protein are expected due to Ntsr1 being expressed in *E. coli*, which lacks the post‐translational modifications possible in the eukaryotic cells expressing Ntr1 (Gray, 1997).

**FIGURE 8:**
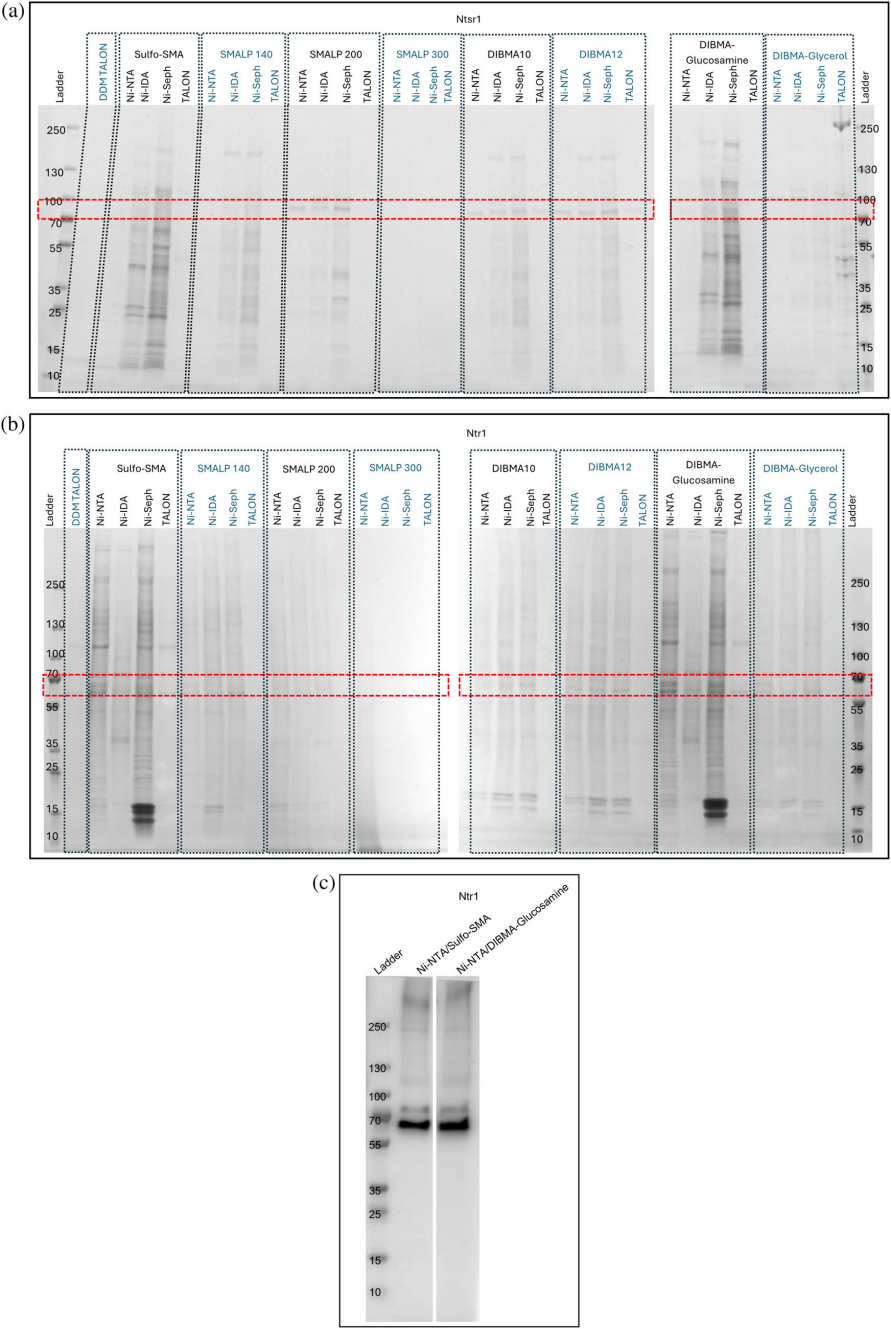
RePol screen results of Ntsr1 (a) and Ntr1 (b and c) resolved on a 4%–15% gradient tris/glycine SDS‐PAGE gel. Ntsr1 and Ntr1 SDS‐PAGE results (a and b) were stained with Coomassie quick stain. Ntr1 (c) was immunoblotted as described in Section 2. All bands visualized on (c) are Ntr1 in various oligomeric or degraded states. DDM solubilized target, extracted using TALON from Takara; Ni‐NTA, Ni‐NTA Superflow from Qiagen; Ni‐IDA, Ni‐IDA from Macherey‐Nagel Protino; Ni‐Seph, Ni‐sepharose high performance from Cytiva; TALON, TALON from Takara.

The RePol screen results for the two targets vary greatly with Ntr1 producing the highest intensity monomeric bands when purified with sulfo‐SMA and DIBMA‐glucosamine (Figure 8a). In contrast, Ntsr1 favored solubilization with the SMALP 200, DIBMA10, and DIBMA12 polymers (Figure 8b). Comparison of the purity of the samples between the two systems also showed variability, with the *E. coli* expressed Ntsr1 having far fewer background contaminants in most conditions than that seen with the HEK293 expressed Ntr1. This may be due to the lower expression levels that can often be seen with HEK293 systems in comparison to *E. coli* expression systems, thereby reducing the competition for resin binding.

For Ntsr1, the Ni‐NTA sepharose HP resin once again was able to produce the highest yields for the target protein; however this is at the expense of purity. For Ntsr1 the Ni‐NTA sepharose SF and Ni‐IDA conditions both showed reduced background contaminants, with a similarly intense band for the target protein as seen with the Ni‐ NTA sepharose HP resin for its favored polymers, with the remaining polymer conditions failing to be purified with Ni‐NTA sepharose SF. The difference in purity observed between the Ni‐NTA sepharose SF and Ni‐NTA sepharose HP resin is interesting as both use the same chelating group and support material (nitrilotriacetic acid and sepharose in both cases). The only difference is the resin bead size, with Ni‐NTA sepharose SF having a larger bead size than that of Ni‐NTA sepharose HP. Why there is a difference in purity is difficult to discern from this data alone, but it is possible that the different bead sizes and therefore the surface areas in the set volume of resins used have an influence on the elutions.

For Ntr1, the Ni‐NTA sepharose SF and Ni‐NTA sepharose HP resin under the sulfo‐SMA and DIBMA‐glucosamine conditions produced the most intense monomeric band, with the main difference being the presence of three strong low molecular weight bands seen in line with the 15 kDa ladder marker. These are present with the Ni‐NTA sepharose HP resin and are not with the Ni‐NTA sepharose SF resin. A Western blot was done on the sulfo‐SMA Ni‐NTA sepharose SF and DIBMA‐glucosamine Ni‐NTA sepharose SF conditions (Figure 8c). Band densitometry analysis showed that the total abundance of Ntr1 is greater with the DIBMA‐glucosamine Ni‐NTA sepharose SF condition than the sulfo‐SMA Ni‐NTA sepharose SF condition (Ntr1 abundance in the sulfo‐SMA Ni‐NTA sepharose SF condition being only 89.7% relative to the DIBMA‐glucosamine Ni‐NTA sepharose SF condition), and purifies a higher abundance of the monomer (89.4% vs. 87.7% monomer for DIBMA‐glucosamine Ni‐NTA sepharose SF condition and sulfo‐SMA Ni‐NTA sepharose SF condition respectively). The presence of higher order oligomers for GPCRs has previously been documented (Jae Ryoung et al., 2010), and is once again seen here with the Western blot analysis. Even with denaturing PAGE conditions the presence of any oligomers is not unprecedented when it comes to integral membrane protein targets due to their propensity to retain some structure in detergents like SDS (Watt et al., 2013) For the mammalian expressed Ntr1, the condition with the least background to the protein of interest is the DIBMA10 Ni‐IDA and DIBMA10 Ni‐NTA sepharose HP. This therefore may be the optimal choice for this target, compromising the abundance of target protein for the best background to target ratio as it has fewer contaminants relative to sulfo‐SMA and DIBMA‐glucosamine.

For low expressing targets Western blot analysis is possible for the whole screen. This was done as an example for Ntsr1 and is included in Figure S2. SMALP 300 was not included due to the lack of any bands observed on the SDS‐PAGE gel. If partnered with SMA‐PAGE, this form of analysis could also highlight how certain polymers and resin combinations may favor different oligomeric states of the target.

## 4 DISCUSSION

Here we report the development of a high throughput screen that enables researchers to efficiently select optimal polymer/chromatography resin combinations for membrane protein extraction and purification. While this approach was capable of rapidly identifying optimal conditions it was unable to identify “rules” that could guide the use of these materials for membrane protein production. However, it may be possible to consider minimal “rules” for the selection of polymer/resin combinations that require the support of screening methodologies such as described here. We show that the use of TALON resin is generally not optimal under the conditions tested, and that at least for this commercial screen, the 3:1 SMA is not effective. This contrasts with previous studies (Morrison et al., 2016; Swainsbury et al., 2017; Unger et al., 2021) and highlights the need for further study to investigate the supply and/or formulation of these reagents. A flowchart (see Figure S4) has been made to simplify the selection process.

SMALP300 from various sources was studied, including two batches of SMALP 300 from Cube Biotech, a sample of SZ25010 from Polyscope and a sample of SMA3000 from Cray Valley. The behavior of the two samples from Cube Biotech matches that seen within this study; however, the Cray Valley and Polyscope equivalents do not. Twenty‐four hours (18 h at 4°C followed by 6 h at room temperature) of trying to dissolve the SMALP300 from Cube Biotech in solution resulted in only partial success, with much polymer remaining out of solution. However, the Cray Valley and Polyscope equivalents were able to fully dissolve (see Image S1a). When an isolated membrane fraction was then solubilized with the various SMALP300 co‐polymers, in line with the methodology highlighted in this paper, considerably more aggregate was visible by eye from using the Cube Biotech polymer in relation to the Cray Valley or Polyscope equivalent (see Image S1b).

Out of the four resins tested, TALON resin was the least successful in capturing any proteins in synthetic polymer nanodiscs; however it was more often than not successful when the target protein was in a DDM micelle, with only Ntsr1 showing no bands in the expected region on the gel in the DDM TALON condition. Only for KcsA was DDM/TALON shown to be better than any of the other conditions used in this screen.

It is well known that cobalt‐based resins have higher specificity but lower affinity than their nickel counterparts. However, the stark difference seen between the TALON resin and nickel‐based resins used in this study seems to fall outside the expected range of these characteristics. To test this, the Ni‐sepharose high performance resin was stripped of its nickel ions and recharged with cobalt ions as outlined in Section 2. The same process was attempted for the TALON resin by Takara, with the aim of recharging it with nickel ions. This process was unsuccessful as the attempts to strip the cobalt from the resin failed, even with increased concentrations of reducing agent (up to 1 M EDTA in distilled water). Upon repeating the RePol screening methodology using the PgsA construct solubilized in SMALP 200 from the Cube Biotech Synthetic Nanodisc Screening Kit MINI the results showed characteristics that were more in line with the expected outcome (see Figure S3a,b). The cobalt‐charged resin exhibited a lower affinity for the target protein, resulting in greater loss during the wash steps, producing a smaller abundance of PgsA in the final elution but with a cleaner background in comparison to the nickel‐charged equivalent. This differs greatly from what we saw with the TALON resin from Takara, where there was no protein at all present in the elution. The TALON resin shares similar properties to the cobalt‐charged Ni‐sepharose high performance resin, with the main difference being the chelating agent, suggesting that carboxymethylated aspartate is partially involved with the lack of binding. This, in combination with cobalt‐based resins requiring a much stricter spatial orientation of the histidines within a poly‐his tag (Team TBB, 2019), may further decrease the affinity of TALON resin to a polyhistidine tag.

From the data we can see that the ZipA construct had the most affinity with TALON resin in comparison to all other targets, as some level of purification was possible with six of the eight polymers. As previously stated, the ZipA construct 6‐his tag is bound to the C‐terminal soluble domain, meaning that it is far from the nanodisc and easily accessible to the affinity resins. All other targets, except PgsA, have the polyhistidine tag relatively close to the membrane bilayer. The PgsA construct, however, has additional tags between the N‐terminus of PgsA and the 9‐His tag, potentially imparting some flexibility, allowing the 9‐his tag to fold and be blocked by the nanodisc.

It has previously been shown that in mildly basic conditions, such as the buffer used in this screen, the charge of the imidazole side chain of histidine, that is, the main interacting partner to the IMAC resins, becomes neutral (Rötzschke et al., 2002). It would not be unfeasible for this to in turn interact with the hydrophobic regions of the nanodisc, sterically hindering the accessibility of the poly‐histidine tag. Furthermore, the acidic subunit in the tested polymers may protonate any imidazole side group of the histidine tags if in close enough proximity, such as those seen with all targets except ZipA. The protonation of the imidazole inhibits any interactions between the bound metal ions in the IMAC resin and the histidine in the polyhistidine tag (Liao et al., 2013). To test this, the pH of all of the buffers was increased to 8.0, increasing the availability of hydroxide ions in an attempt to reduce the protonation of the imidazole side group. PgsA was then re‐screened under the SMALP 200/TALON condition. This resulted in a distinct band in the expected region of the gel, showing a substantial increase in affinity of the poly‐histidine tag to the resin (see Figure S3c). This suggests that the pH sensitivity of the poly‐histidine tag, in combination with the lower affinity and the strict spatial requirements of cobalt bound to a carboxymethylated aspartate chelating agent, may be the reason why poly‐histidine tags too close to the synthetic nanodisc fail to bind to TALON resin. To confirm the validity of this hypothesis, additional experimental investigations are warranted.

We also show that across the proteins screened in this study, in general, the best performing resin was one that used NTA as the nickel chelating group. These data also showed that even with the same chelating group, for some proteins (e.g., Ntsr1) subtle alterations in the matrix on which the chelating group was presented could alter the purity of the protein. This shows that a combined polymer/resin screening strategy is important if optimal purification conditions are to be discovered.

Our screening method showed that even for proteins with high sequence identity (and hence structure), optimal conditions differed markedly. This may derive from a number of factors including interactions of polymers with the local bilayer around the chosen protein and even interaction of the polymer with the protein. Additionally, once the disc has formed, interaction of the disc with the resin could be variable as is how the disc influences the position of the purification tag. In summary, this study demonstrates the benefit of a 2‐day high‐throughput approach to developing polymer‐based strategies for the purification of membrane proteins that can be highly personalized, scalable and automatable using modern equipment such as the CyBio FeliX system from Analytik Jena coupled with the PhyTip® Columns from Biotage®. This methodology is not only compatible with the premade polymer screen used in this study, but with any polymer set that the user desires. A flowchart to aid in deciding the optimal condition for the user's application has been included in Figure S4. Our study provides a resource for the community to build upon in the exploration of SMA technology and detergent‐free extraction and analysis of membrane protein structure and function.

## AUTHOR CONTRIBUTIONS

**Adam Evans:** Conceptualization; investigation; methodology; writing – review and editing; data curation; writing – original draft; formal analysis. **Bethan Kelly:** Resources; writing – review and editing. **Pooja Sridhar:** Resources; writing – review and editing. **Alice J. Rothnie:** Resources; writing – review and editing. **Naomi L. Pollock:** Resources; writing – review and editing. **Philip M. Ireland:** Resources; writing – review and editing; validation; funding acquisition; supervision; formal analysis. **David I. Roper:** Funding acquisition; writing – review and editing; validation; resources; supervision; formal analysis. **Tim R. Dafforn:** Funding acquisition; writing – review and editing; validation; resources; formal analysis; supervision.

## Supporting information


**IMAGE S1.** (a) The difference in dissolvability between the batches of Cube Biotech SMALP 300 and the Cray valley and Polyscope equivalent (far left—Cube Biotech; middle left—Cube Biotech; middle right—Cray Valley; far right—Polyscope). (b) The difference in membrane solubilization between the batches of Cube Biotech SMALP300 and the Cray Valley and Polyscope equivalent (far left—Cube Biotech; middle left—Cube Biotech; middle right—Cray Valley; far right—Polyscope).

**FIGURE S1.** (a) The band densitometry results for the relative percentage of KcsA across all conditions. The numerical value is written above the bars, with the SDS‐PAGE below showing the detected bands. (b) The band densitometry results for the relative abundance of KcsA within each condition. The numerical value written above the bars shows the percentage value that each band contributes to that condition, with an asterisk identifying the KcsA band. The SDS‐PAGE to the left of each graph is annotated, showing the detected bands. Band densitometry was determined using ImageJ 1.54g software. Ni‐IDA, Ni‐IDA from Macherey‐Nagel Protino; Ni‐NTA, Ni‐NTA Superflow from Qiagen; Ni‐Seph, Ni‐sepharose high performance from Cytiva; TALON, TALON from Takara.

**FIGURE S2.** RePol screen results of Ntsr1 resolved on a 4%–15% gradient tris/glycine SDS‐PAGE gel and was immunoblotted as described in the methods. The red arrow is used to indicate the monomer of Ntsr1. Ni‐IDA, Ni‐IDA from Macherey‐Nagel Protino; Ni‐NTA, Ni‐NTA Superflow from Qiagen; Ni‐Seph, Ni‐sepharose high performance from Cytiva; TALON, TALON from Takara.

**FIGURE S3.** (a) The unbound and wash flowthrough fractions of PgsA solubilized in SMALP 200 when bound to cobalt charged sepharose high performance resin (co‐sepharose) and nickel charged sepharose high performance resin (Ni‐sepharose). Samples were run on a 4%–15% gradient tris/glycine SDS‐PAGE gel stained with Coomassie quick stain. The red dotted box highlights the expected region on the gel that PgsA is expected to migrate to. (b) The eluted fractions of PgsA solubilized in SMALP 200 when bound to cobalt charged sepharose high performance resin (co‐sepharose) and nickel charged sepharose high performance resin (Ni‐sepharose). Samples were run on a 4%–15% gradient tris/glycine SDS‐PAGE gel stained with Coomassie quick stain. The red dotted box highlights the expected region on the gel that PgsA is expected to migrate to. (c) The eluted fractions of PgsA solubilized in SMALP 200 when bound to TALON resin when screened in pH 8.0 buffers. Sample was run on a 4%–15% gradient tris/glycine SDS‐PAGE gel stained with Coomassie quick stain. The red dotted box highlights the expected region on the gel that PgsA is expected to migrate to.

**FIGURE S4.** A simplified flow chart on how to select the optimal condition for the protein of interest.

## Data Availability

The data that support the findings of this study are available from the corresponding author upon reasonable request.

## APPENDIX A

Manufacturer names and addresses for brand products.

**Table:**

Manufacturer	Address	Product
Axygen Inc. Corning	33210 Central Ave, Union City, California, USA	Deep well plate, 24 well
Cube Biotech	Monheim am Rhein, Germany	Synthetic Nanodisc Screening Kit MINI
Avantor	Pennsylvania, USA	Deep well plates square well, V‐bottom
Molecular Dimensions	Sheffield, UK	EasySeal™ Sheets
Pall Life Sciences	New York, USA	96 Well Filter Plate Acroprep Advance 2 mL 0.2 μm PTFE Membrane
Invitrogen	California, USA	NuPAGE™ LDS Sample Buffer (4X)
BioRad	California, USA	4–15% Criterion™ TGX™ Precast Midi Protein Gel, 26 wells
BioRad	California, USA	Trans‐Blot Turbo Transfer System and Trans‐Blot Turbo Midi 0.2 μm PVDF Transfer Packs
Qiagen	North Rhine‐Westphalia, Germany	Ni‐NTA Superflow
Macherey‐Nagel	North Rhine‐Westphalia, Germany	Protino Ni‐IDA
Cytiva	Massachusetts, US	Ni Sepharose™ High Performance
Takara	Shiga, Japan	TALON
Polyscope	Limburg, Netherlands	SZ25010
Cray Valley	Moselle, France	SMA3000
Analytik Jena	Thuringia, Germany	CyBio FeliX
Biotage	Uppsala, Sweden	PhyTip® Columns
