# Supplementary figures and images for: RePol: A high‐throughput screen for optimizing membrane protein solubilization and purification using polymers
Source: Protein Sci. 2025 Dec 22;35(1):e70407. doi: 10.1002/pro.70407 (PMC12720780; doi:10.1002/pro.70407)

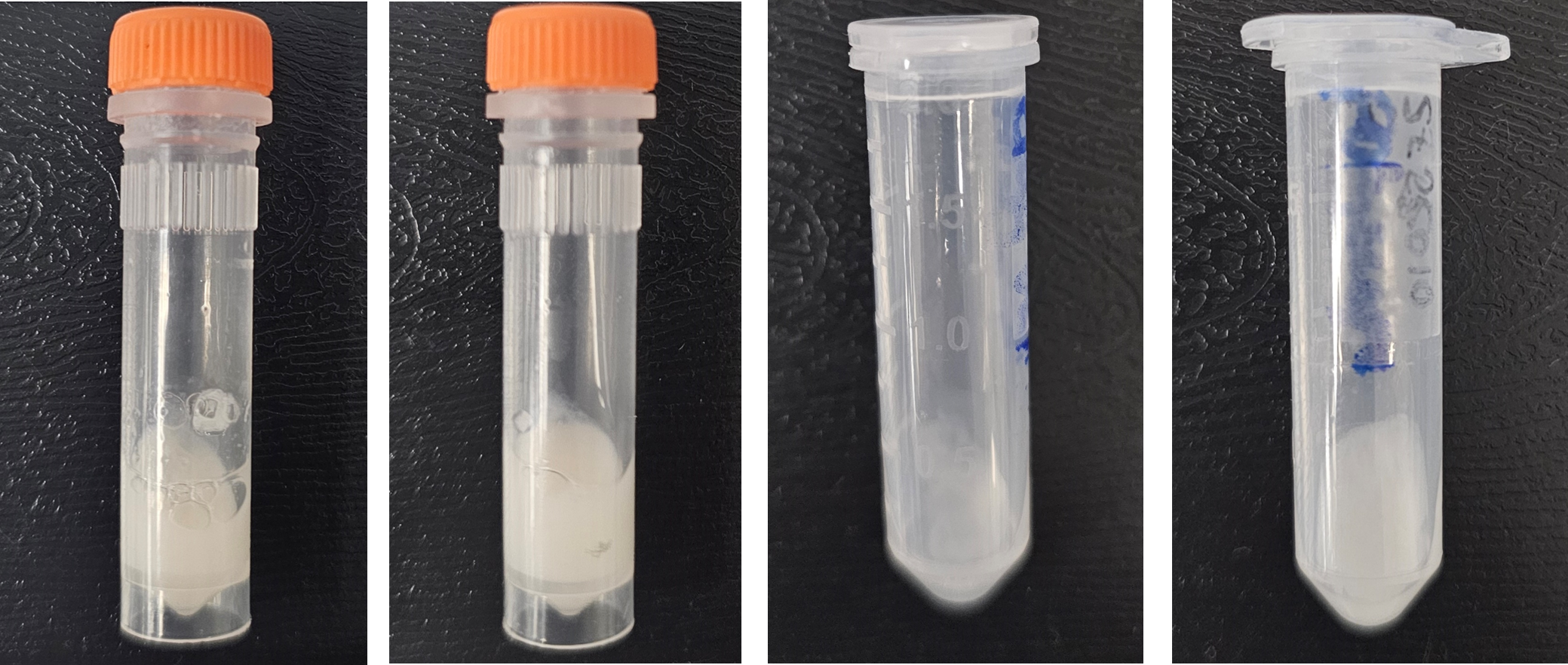

Supplement: Supplementary file 1 — IMAGE S1. (a) The difference in dissolvability between the batches of Cube Biotech SMALP 300 and the Cray valley and Polyscope equivalent (far left—Cube Biotech; middle left—Cube Biotech; middle right—Cray Valley; far right—Polyscope). (b) The difference in membrane solubilization between the batches of Cube Biotech SMALP300 and the Cray Valley and Polyscope equivalent (far left—Cube Biotech; middle left—Cube Biotech; middle right—Cray Valley; far right—Polyscope). [file PRO-35-e70407-s003.zip › PRO_70407_f1_Supplementary image 1b Membrane solubilsation between Cube Biotech, Cray Valley and Polyscope.tif]

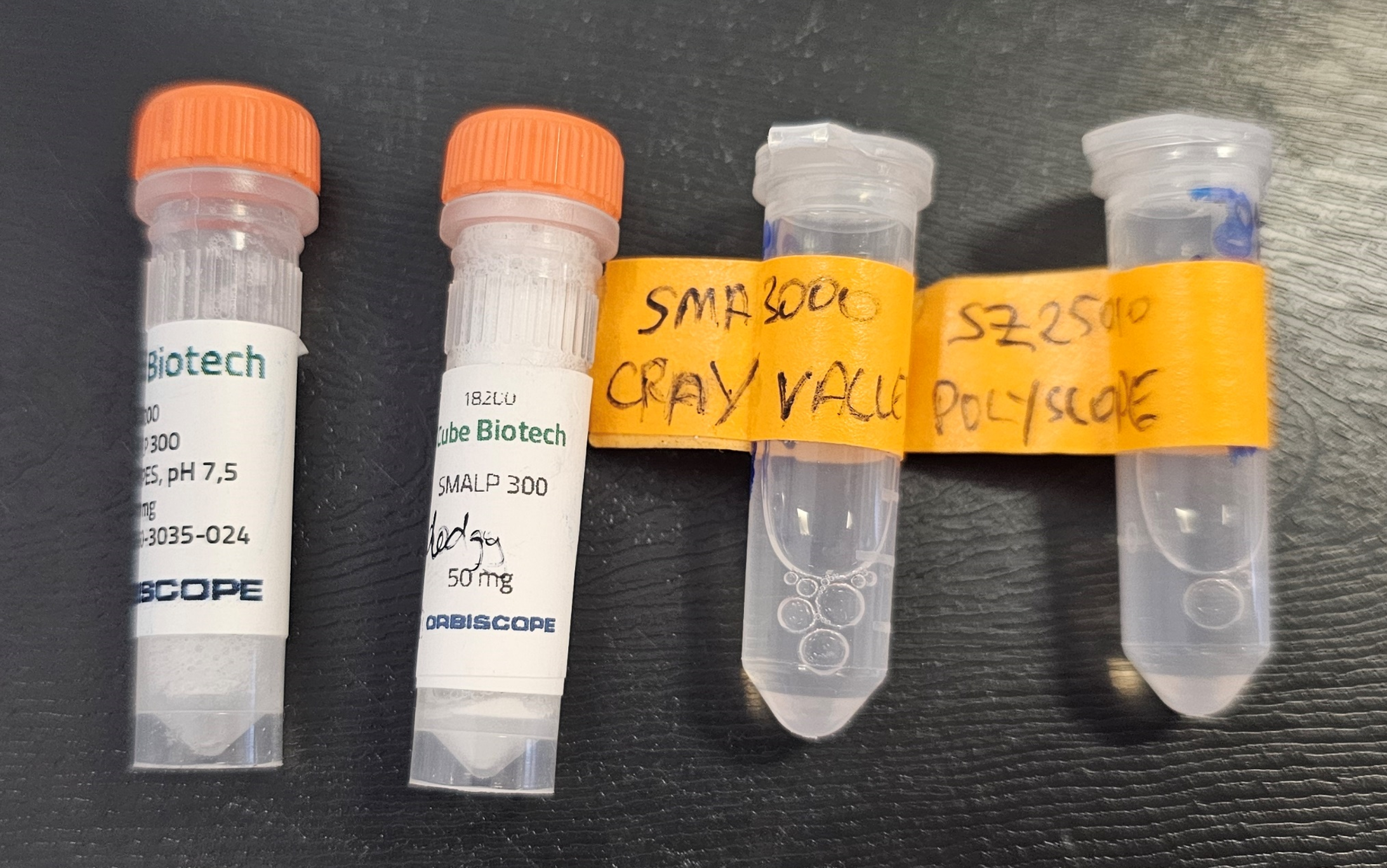

Supplement: Supplementary file 1 — IMAGE S1. (a) The difference in dissolvability between the batches of Cube Biotech SMALP 300 and the Cray valley and Polyscope equivalent (far left—Cube Biotech; middle left—Cube Biotech; middle right—Cray Valley; far right—Polyscope). (b) The difference in membrane solubilization between the batches of Cube Biotech SMALP300 and the Cray Valley and Polyscope equivalent (far left—Cube Biotech; middle left—Cube Biotech; middle right—Cray Valley; far right—Polyscope). [file PRO-35-e70407-s003.zip › PRO_70407_f1_Supplementary image 1a Dissolvability between Cube Biotech, Cray Valley and Polyscope SMA.tif]

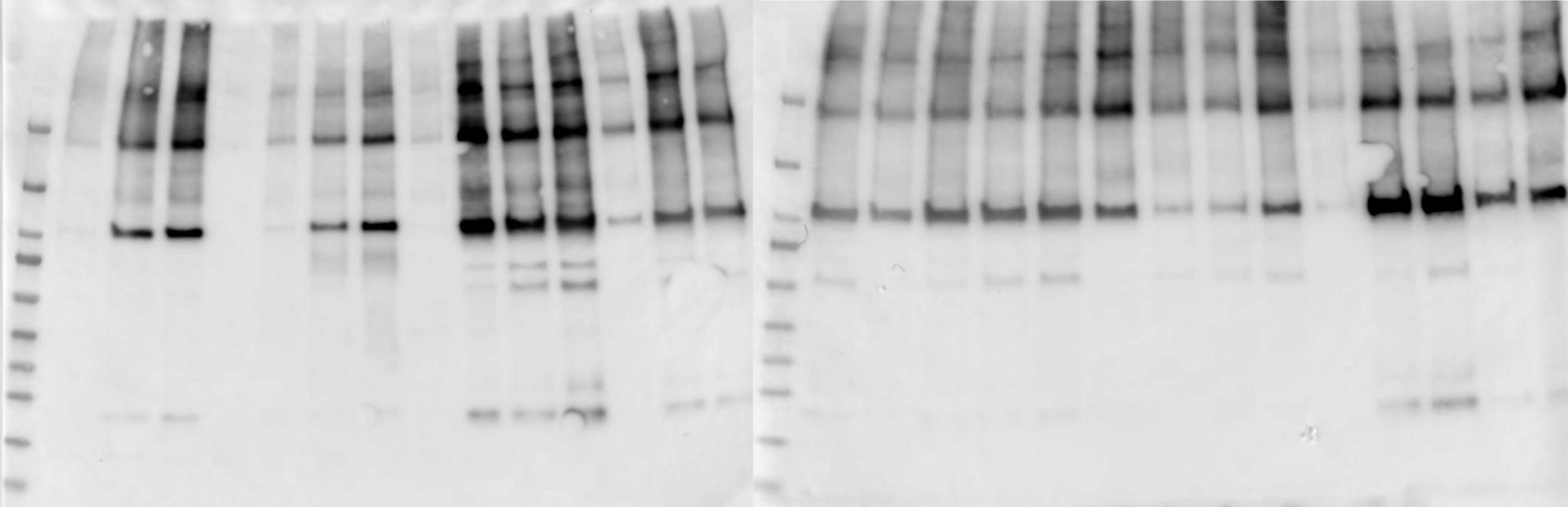

Supplement: Supplementary file 2 — FIGURE S1. (a) The band densitometry results for the relative percentage of KcsA across all conditions. The numerical value is written above the bars, with the SDS‐PAGE below showing the detected bands. (b) The band densitometry results for the relative abundance of KcsA within each condition. The numerical value written above the bars shows the percentage value that each band contributes to that condition, with an asterisk identifying the KcsA band. The SDS‐PAGE to the left of each graph is annotated, showing the detected bands. Band densitometry was determined using ImageJ 1.54g software. Ni‐IDA, Ni‐IDA from Macherey‐Nagel Protino; Ni‐NTA, Ni‐NTA Superflow from Qiagen; Ni‐Seph, Ni‐sepharose high performance from Cytiva; TALON, TALON from Takara. [file PRO-35-e70407-s005.zip › pro70407-sup-0003-FigureS1-S2@Supplementary figure 2 Ntsr1 Western blott.tif]

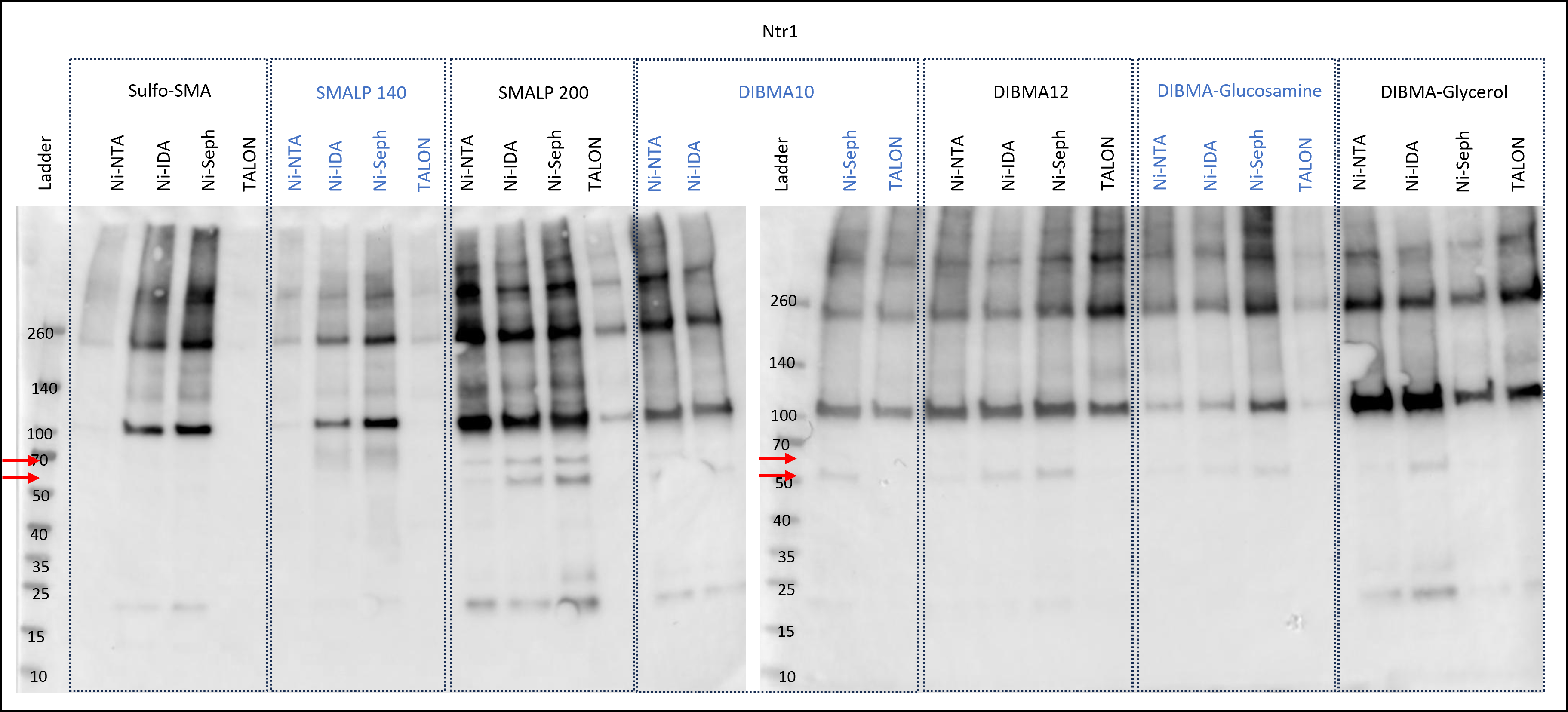

Supplement: Supplementary file 3 — FIGURE S2. RePol screen results of Ntsr1 resolved on a 4%–15% gradient tris/glycine SDS‐PAGE gel and was immunoblotted as described in the methods. The red arrow is used to indicate the monomer of Ntsr1. Ni‐IDA, Ni‐IDA from Macherey‐Nagel Protino; Ni‐NTA, Ni‐NTA Superflow from Qiagen; Ni‐Seph, Ni‐sepharose high performance from Cytiva; TALON, TALON from Takara. [file PRO-35-e70407-s001.tif]

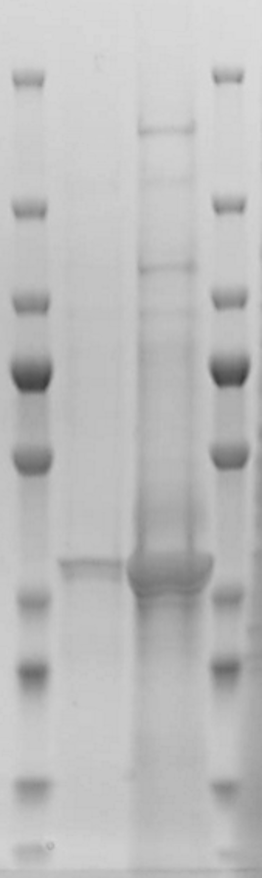

Supplement: Supplementary file 4 — FIGURE S3. (a) The unbound and wash flowthrough fractions of PgsA solubilized in SMALP 200 when bound to cobalt charged sepharose high performance resin (co‐sepharose) and nickel charged sepharose high performance resin (Ni‐sepharose). Samples were run on a 4%–15% gradient tris/glycine SDS‐PAGE gel stained with Coomassie quick stain. The red dotted box highlights the expected region on the gel that PgsA is expected to migrate to. (b) The eluted fractions of PgsA solubilized in SMALP 200 when bound to cobalt charged sepharose high performance resin (co‐sepharose) and nickel charged sepharose high performance resin (Ni‐sepharose). Samples were run on a 4%–15% gradient tris/glycine SDS‐PAGE gel stained with Coomassie quick stain. The red dotted box highlights the expected region on the gel that PgsA is expected to migrate to. (c) The eluted fractions of PgsA solubilized in SMALP 200 when bound to TALON resin when screened in pH 8.0 buffers. Sample was run on a 4%–15% gradient tris/glycine SDS‐PAGE gel stained with Coomassie quick stain. The red dotted box highlights the expected region on the gel that PgsA is expected to migrate to. [file PRO-35-e70407-s002.zip › pro70407-sup-0007-FigureS3-S200@Supplementary figure 3b SMALP 200 solubilised PgsA Elutions SDS-PAGE.tif]

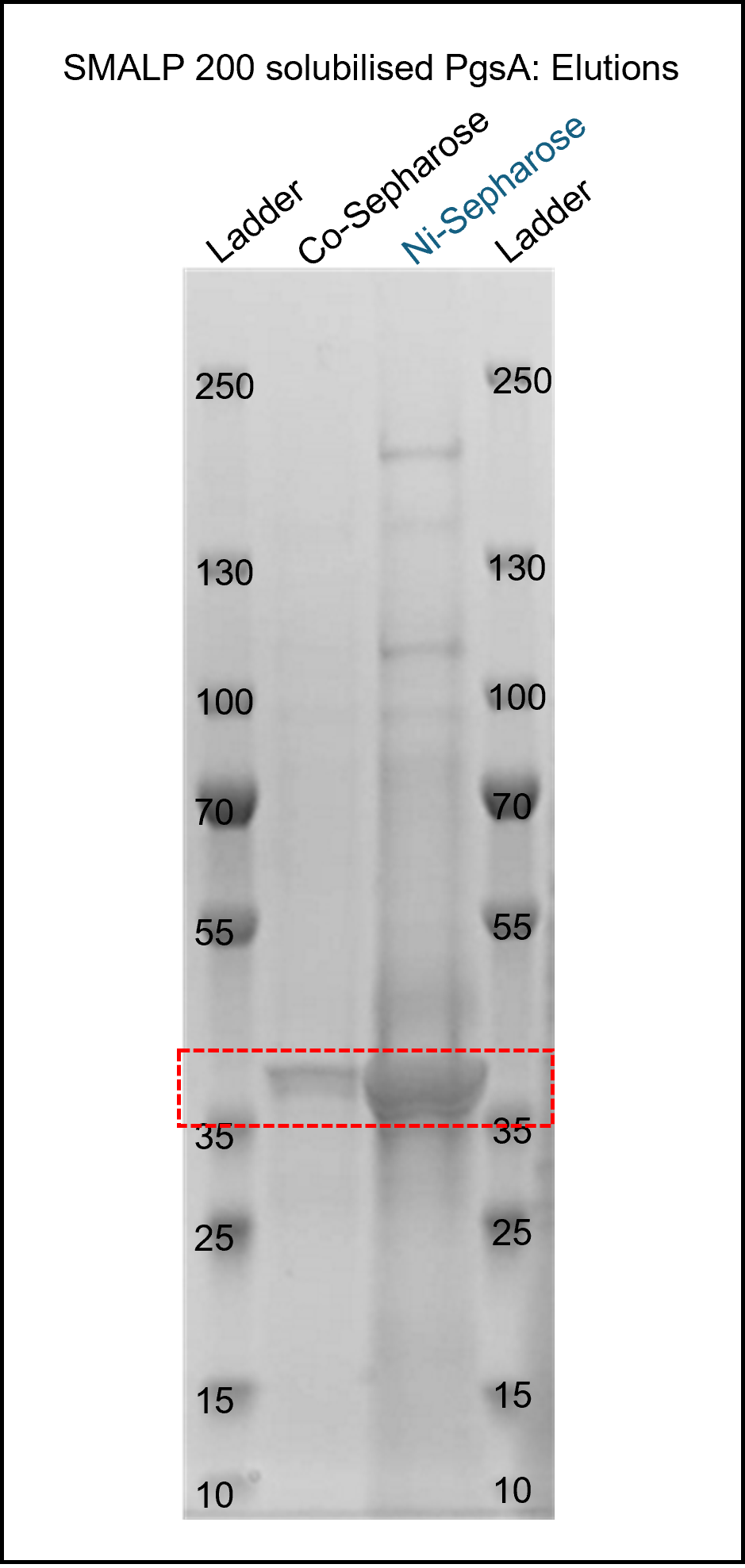

Supplement: Supplementary file 4 — FIGURE S3. (a) The unbound and wash flowthrough fractions of PgsA solubilized in SMALP 200 when bound to cobalt charged sepharose high performance resin (co‐sepharose) and nickel charged sepharose high performance resin (Ni‐sepharose). Samples were run on a 4%–15% gradient tris/glycine SDS‐PAGE gel stained with Coomassie quick stain. The red dotted box highlights the expected region on the gel that PgsA is expected to migrate to. (b) The eluted fractions of PgsA solubilized in SMALP 200 when bound to cobalt charged sepharose high performance resin (co‐sepharose) and nickel charged sepharose high performance resin (Ni‐sepharose). Samples were run on a 4%–15% gradient tris/glycine SDS‐PAGE gel stained with Coomassie quick stain. The red dotted box highlights the expected region on the gel that PgsA is expected to migrate to. (c) The eluted fractions of PgsA solubilized in SMALP 200 when bound to TALON resin when screened in pH 8.0 buffers. Sample was run on a 4%–15% gradient tris/glycine SDS‐PAGE gel stained with Coomassie quick stain. The red dotted box highlights the expected region on the gel that PgsA is expected to migrate to. [file PRO-35-e70407-s002.zip › pro70407-sup-0008-FigureS3@Supplementary Figure 3b.tif]

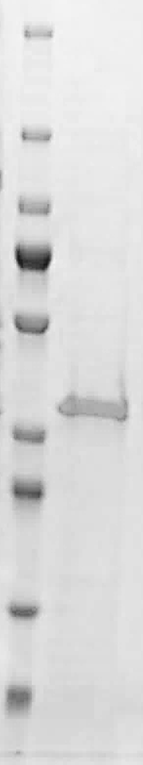

Supplement: Supplementary file 4 — FIGURE S3. (a) The unbound and wash flowthrough fractions of PgsA solubilized in SMALP 200 when bound to cobalt charged sepharose high performance resin (co‐sepharose) and nickel charged sepharose high performance resin (Ni‐sepharose). Samples were run on a 4%–15% gradient tris/glycine SDS‐PAGE gel stained with Coomassie quick stain. The red dotted box highlights the expected region on the gel that PgsA is expected to migrate to. (b) The eluted fractions of PgsA solubilized in SMALP 200 when bound to cobalt charged sepharose high performance resin (co‐sepharose) and nickel charged sepharose high performance resin (Ni‐sepharose). Samples were run on a 4%–15% gradient tris/glycine SDS‐PAGE gel stained with Coomassie quick stain. The red dotted box highlights the expected region on the gel that PgsA is expected to migrate to. (c) The eluted fractions of PgsA solubilized in SMALP 200 when bound to TALON resin when screened in pH 8.0 buffers. Sample was run on a 4%–15% gradient tris/glycine SDS‐PAGE gel stained with Coomassie quick stain. The red dotted box highlights the expected region on the gel that PgsA is expected to migrate to. [file PRO-35-e70407-s002.zip › pro70407-sup-0009-FigureS3-S200@Supplementary figure 3c SMALP 200 solubilised PgsA pH 8 SDS-PAGE.tif]

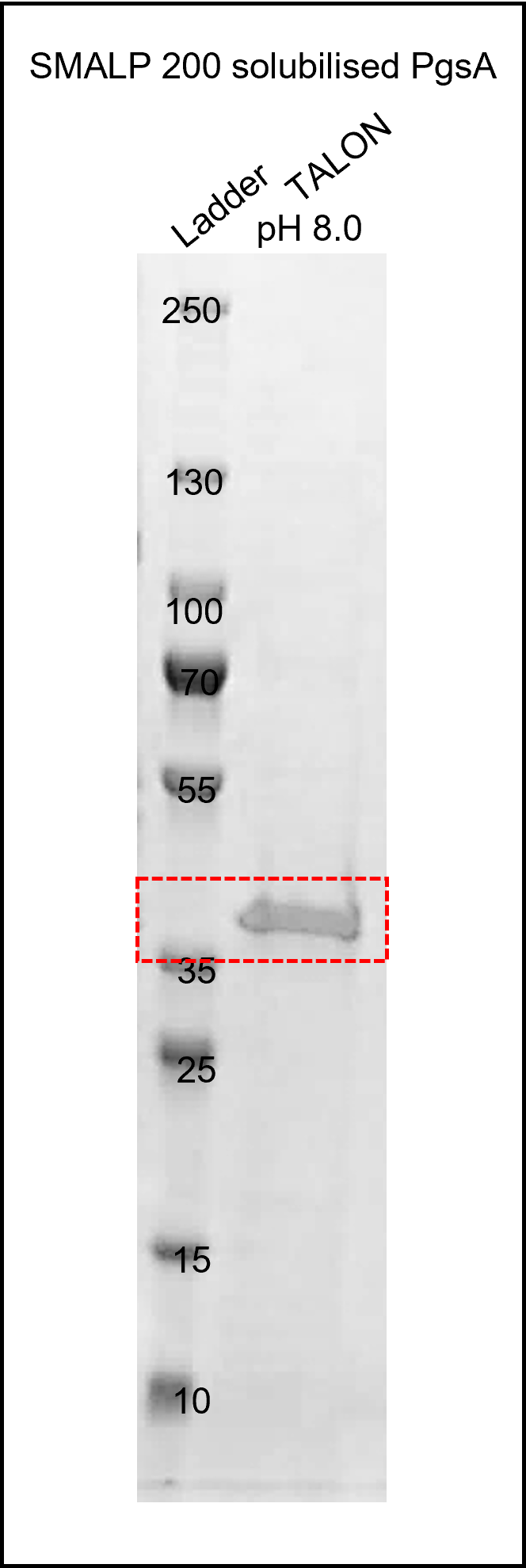

Supplement: Supplementary file 4 — FIGURE S3. (a) The unbound and wash flowthrough fractions of PgsA solubilized in SMALP 200 when bound to cobalt charged sepharose high performance resin (co‐sepharose) and nickel charged sepharose high performance resin (Ni‐sepharose). Samples were run on a 4%–15% gradient tris/glycine SDS‐PAGE gel stained with Coomassie quick stain. The red dotted box highlights the expected region on the gel that PgsA is expected to migrate to. (b) The eluted fractions of PgsA solubilized in SMALP 200 when bound to cobalt charged sepharose high performance resin (co‐sepharose) and nickel charged sepharose high performance resin (Ni‐sepharose). Samples were run on a 4%–15% gradient tris/glycine SDS‐PAGE gel stained with Coomassie quick stain. The red dotted box highlights the expected region on the gel that PgsA is expected to migrate to. (c) The eluted fractions of PgsA solubilized in SMALP 200 when bound to TALON resin when screened in pH 8.0 buffers. Sample was run on a 4%–15% gradient tris/glycine SDS‐PAGE gel stained with Coomassie quick stain. The red dotted box highlights the expected region on the gel that PgsA is expected to migrate to. [file PRO-35-e70407-s002.zip › pro70407-sup-0010-FigureS3@Supplementary Figure 3c.tif]

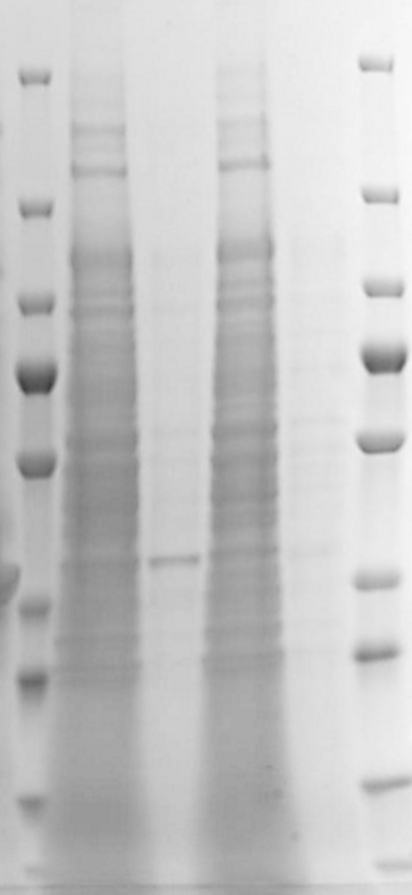

Supplement: Supplementary file 4 — FIGURE S3. (a) The unbound and wash flowthrough fractions of PgsA solubilized in SMALP 200 when bound to cobalt charged sepharose high performance resin (co‐sepharose) and nickel charged sepharose high performance resin (Ni‐sepharose). Samples were run on a 4%–15% gradient tris/glycine SDS‐PAGE gel stained with Coomassie quick stain. The red dotted box highlights the expected region on the gel that PgsA is expected to migrate to. (b) The eluted fractions of PgsA solubilized in SMALP 200 when bound to cobalt charged sepharose high performance resin (co‐sepharose) and nickel charged sepharose high performance resin (Ni‐sepharose). Samples were run on a 4%–15% gradient tris/glycine SDS‐PAGE gel stained with Coomassie quick stain. The red dotted box highlights the expected region on the gel that PgsA is expected to migrate to. (c) The eluted fractions of PgsA solubilized in SMALP 200 when bound to TALON resin when screened in pH 8.0 buffers. Sample was run on a 4%–15% gradient tris/glycine SDS‐PAGE gel stained with Coomassie quick stain. The red dotted box highlights the expected region on the gel that PgsA is expected to migrate to. [file PRO-35-e70407-s002.zip › pro70407-sup-0005-FigureS3-S200@Supplementary figure 3a SMALP 200 solubilised PgsA Unbound and wash fractions SDS-PAGE.tif]

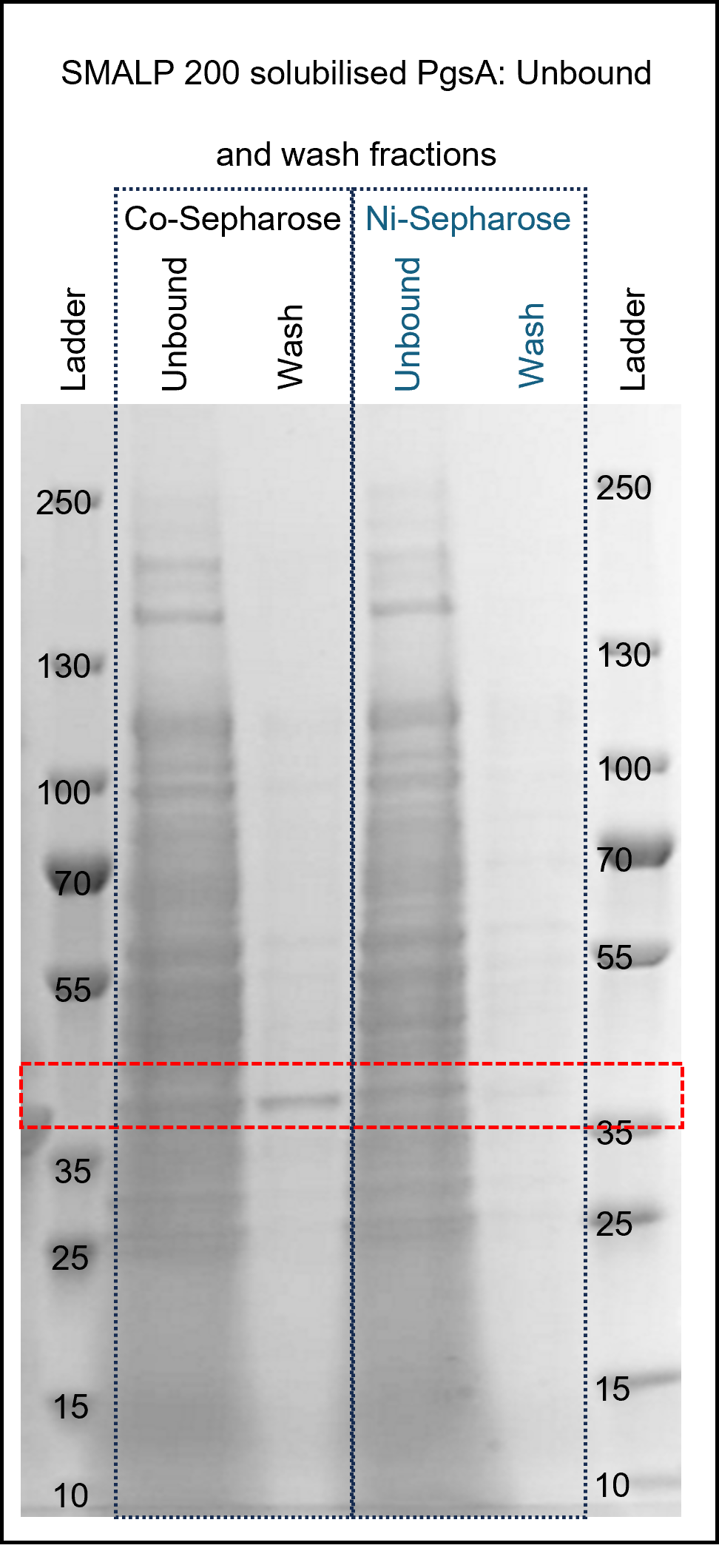

Supplement: Supplementary file 4 — FIGURE S3. (a) The unbound and wash flowthrough fractions of PgsA solubilized in SMALP 200 when bound to cobalt charged sepharose high performance resin (co‐sepharose) and nickel charged sepharose high performance resin (Ni‐sepharose). Samples were run on a 4%–15% gradient tris/glycine SDS‐PAGE gel stained with Coomassie quick stain. The red dotted box highlights the expected region on the gel that PgsA is expected to migrate to. (b) The eluted fractions of PgsA solubilized in SMALP 200 when bound to cobalt charged sepharose high performance resin (co‐sepharose) and nickel charged sepharose high performance resin (Ni‐sepharose). Samples were run on a 4%–15% gradient tris/glycine SDS‐PAGE gel stained with Coomassie quick stain. The red dotted box highlights the expected region on the gel that PgsA is expected to migrate to. (c) The eluted fractions of PgsA solubilized in SMALP 200 when bound to TALON resin when screened in pH 8.0 buffers. Sample was run on a 4%–15% gradient tris/glycine SDS‐PAGE gel stained with Coomassie quick stain. The red dotted box highlights the expected region on the gel that PgsA is expected to migrate to. [file PRO-35-e70407-s002.zip › pro70407-sup-0006-FigureS3@Supplementary Figure 3a.tif]

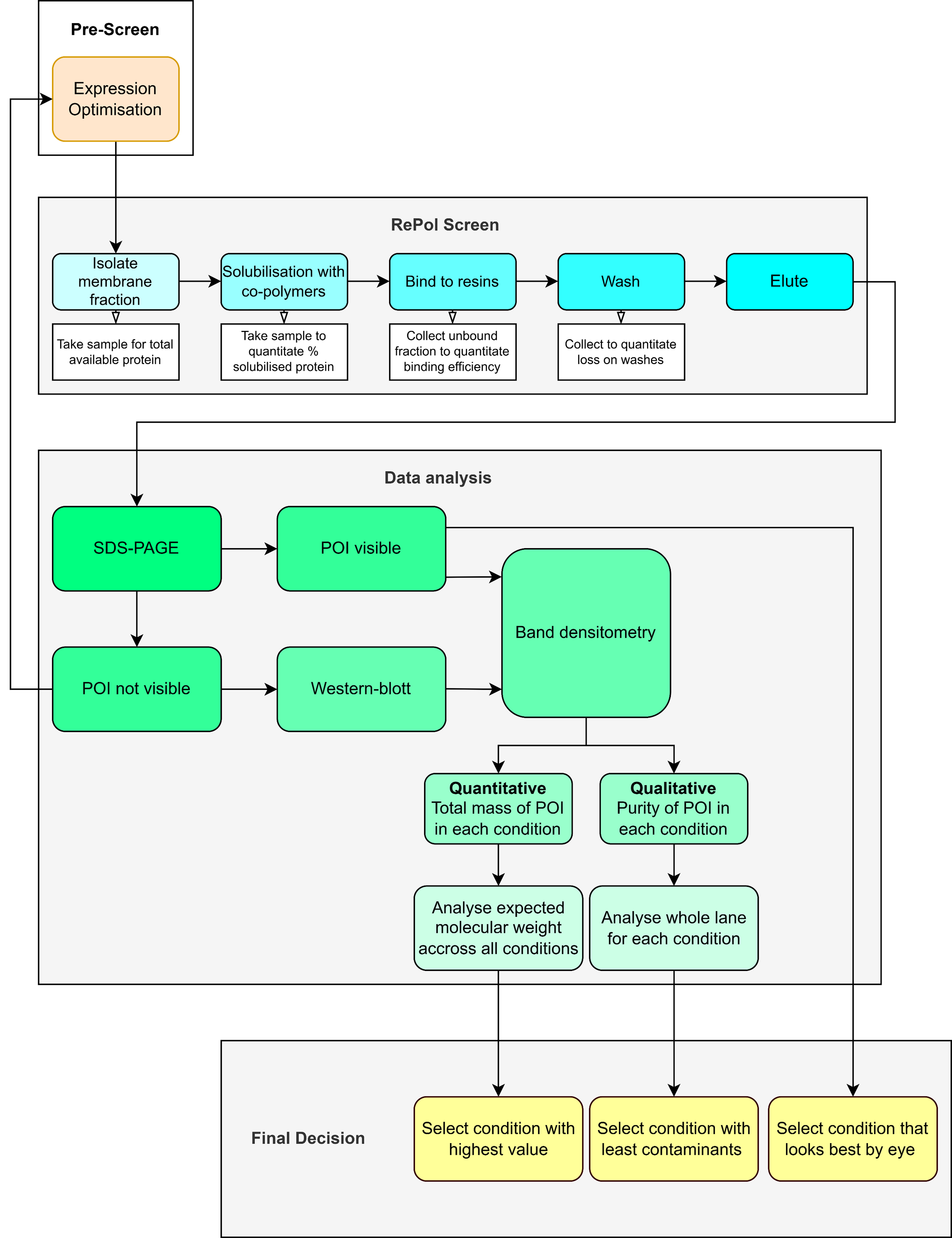

Supplement: Supplementary file 5 — FIGURE S4. A simplified flow chart on how to select the optimal condition for the protein of interest. [file PRO-35-e70407-s004.tif]
